# Supplementary material for: EternaBrain: Automated RNA design through move sets and strategies from an Internet-scale RNA videogame
Source: PLoS Comput Biol. 2019 Jun 27;15(6):e1007059. doi: 10.1371/journal.pcbi.1007059 (PMC6597038; doi:10.1371/journal.pcbi.1007059)
Supplement: S1 File — Participants who tooks part in solving the puzzles used to develop EternaBrain, with names listed in order of contributions, from most to least. (DOCX) [file pcbi.1007059.s001.docx]

We acknowledge the following Eterna participants for taking part in solving the puzzles used to develop EternaBrain, with names listed in order of contributions, from most to least:

Eli Fisker, dl2007, Pi, DeNa, mat747, HarryS, Malcolm, Jieux, eternacac, JR, benrh, cynwulf28, Omei, Poll na gColm, GFRANK2, JSci, Brourd, spvincent, whbob, tommyd, skyblue, dizzywings, lroppy, AndrewKae, atanas.atanasov, rxmullin, worseize, ElNando888, hoglahoo, holoaaron, 137.036, jandersonlee, Astromon, Brevitz, bjorns, novice, BHunter, chloep17, Tesla'sDisciple, kriss888, Sgtbird08, lcurtisadams, carmenmarbella, pmlkjn, a1of2twin2016, quantropy, JONATHANROHR, RasmusVH, RAH1, Fusion7, Warren09, Gres, ulfang, joy45, Zanna, D'Wydd, garrin, yceev, Arnthi_470, astralcrescent, erwinmulders, MacSousa, garydfisher, AndrewReedy13, sn69, Starna, Gracier, wawan151, ZOONIVERSE12, wateronthemoon, Radioactive7, IceBolt, ererexiue, LarryB, camdenmurray, manzet, armin, ppgbubbles, FurElise, biggestlegoheroicafanever, AnnYang, Snowpup, chase2003, eagercheesecake, Sven4, heynorma, DaIto, jsydrah, dudbomber, incal11, kwharton, fallenwarior, Citizen Science Guy, Sargon, c-quence, LiquidOvar, dblueskye, ZachFR, Shayera, mastorasa, Grelko, scientificdash, Marculius, SockTaters, elasmo, TheDomBom13, jIyHHbIu, davidwhittaker, ecedenia, Ethaniel, kkdixon, ciprian, JoshS5, doofenshmirtz, justintwayland, jbrandim, MeowNow360, delcastle, Pytho, AustonTheriault, Eized, BirchSci25, cjaltrichter, Nirrame, VeraMarsova, CarlosFaustino, billygoat, everyday847, Matias Nicolas, wookieetank, Citizen Bane, ShelleyVAdams, gian666, skamil1204, PaulaSphere, SourCreamKing, ChrisRWitt, tone, annamary488, ljchapa, king1000, Pilot69, sellena, Loreleykaa, tamaki, tmjlowe, danielmcmurray, DarkAndroid, bbueno5000, SalarianScientist, Wulveniel, rchrdjhnsn, Overide, mtor1, henege, Masterbajurf, Leepretorius, janetmason, Tati69, alebec, lindelenilda, dillon101001, kkowales1, Mongoose, rocketdog42, apeter14, Nova631693, Ekymose, chanceyko, RunDMC, Armordines, pamisza, a1puterboy, mzeinstra, dragoncendre, Radek24, Psykovsky, MageOfTime, kiernan2, marcuac, jessesmall, jonah.pandey, booti386, LAdarkTrooper, qq47, D1l3mm4, science123, Zugai, Felixpi, Aerendil, PereKastor, 200611736, mjmtl, akhyatt, mircea, Suboptimalautomation, Menosfree, smwalsh1228, StormTroop, Sylva, trashy, Jeshyr, SolubleCarpet, EcceruElme, RNA123, tklight13, Darkasleif, GermanP, Lewis656, nico1630, bjackblack, TheStaker07, kylelyk, Sean O'Neil, jnicol, Gigante, mariapaglinawan, bryanb2, YenRaven, TAG, ch.parushev@gmail.com, chalouky, onilink_, Tessaract2, MasterStormer, deltaq84, Suprem, adam.g.fischer@gmail.com, David.Sampson@oregonstate.edu, Renthel, awesomehuman, cosmia, cmmtchll, Xnessax, angusd, Ambiseus, nuqotw, Manabender, oli.veri, Lyssaodr, alacarus, chop&toss, XFYLESGIRL20, Trentis1, manthonyd1618, wkdus108, Taflin, Mayanne, lastresortist, xotame, Hyphema, MThrasher0, Baron, Moir, dbm, JulesLeNomad, Soken, Meechl, norikusan, AxelHawke, Jimedney, redrocz, HawkEyesMiHawk, Marzena11, emjay, zsherman, sing146, acebassmaster, Njdilaan, hai-meh, Calanor, kersevich, AshlyW42, Aleks59, freznow, anik, all, taslack, Ipiu, SkyDancer, alecpikachu, SrdjanM, rlhruza, saili6, potato43, pabloguillen, Nsyp, 2016_talias, RayA, Egin, AmyW, sneaky90210, Monsoon, EPogue, Antireta, jahfe, whispywhip, rubixcube517, pyrazole, trili, Willcalliw, sebC, akio123, grass, Cawa, RechleckiJ, aceiestatheist, randomfull9, paÁoqueiro, madman200, PFKThorin, HARDWAREGUY, Ibanezmtl, chuckcoleman, harpokrates, Vincorporated, AHamel, Eceri, KT006, GColdwell, adamsunny, Niemand, marisa31, mrbell, TedStudley, jyoshimi, Approved, IchigoNandato, PenguinLea, stevetclark, bbk123, sharmoni, melliniumfalcon1029, JennTM, Natura, bookbird, Duha Eldow, Teutonius, mdurbin, kartonrealistah, rafa_prog, 12walch, bkopel, TakumaInoue, caro9923, Theos, LordHaralVII, legotourist, ThatYouGoOn, 1900029, theta123, raek57, MysteryBacon, Dragonbard, redkatie, Purple wizard, zombiekill6r, Rouge Lead, 577661, wnxkitchenaid, cutkzig, Sporeo, System0X, sekeerne, Skye, LCL46fgh7, lferris1, Jt82497, scooper1, Happymuffen, Aleksandr, maschiltz, PeakeHaus, Doctor_Maximus, coolbeans55555, dmitrybaianov, Kiddodog, tdmcmillen, LacXav, StuartC, DGFigueiroa, EmeraldDragon, kmille, Paulo Roque, rdfgiraldez, Stephanie1978a, turing999, balsamina, rdaleg3, vdvd138, darkseariver, PIRSQUARED , Marquis Tinkles, stormyx13, rhys, fareedanyc, wallr1, RNADesigner, zacthemac753, kang36, cptguru, Schwarzee, stephancannon, leanicholewhite, Maya are you here, seriousgedas, Abilatanwen, rjonzales, vjayant, adibou_super_saiyan, silent dicer, sheyla, starreus, choo, aclockworkkc, pksvseng, lizdeath, Vorushea, CaptainColon, Billy Reuben, LaMariposa, Bekreth, captainpsychology1, iojp, PFChopZ, jackieofall, Anorectic99, firefliesjr, Professor Lamp, Mathiszenoob, Alec_7, FelisAnxietus, Gwindorzp, microbiogenius, Jack123X, lqtza, tc5801, jgabbott, lilyflan12, hi25114, Abdullah, simonk, funglish, joe911, Ozzy1313, TheDoctorInTheHouse, jcastello, ExternalCPU, zeroxoxo, zakblak, couchkitty, hizowie, Orkhan, arueda, randomone66, Eliisme, player613, nijaho, kyger1, mh.ku, Bpala, dmeyer, dentongal, Andoryuuta, rguioguio, osomblossom, MauroBedoya, Kienan2, Hayfieldlee, hibts, kijizz, lkjhnsn7, bholcombe1, RayNA, serinorah, smuthuganesh, Science Ferret, danryk, cwillms, BletchleyPark, roughb06, Jesse2016, Tigerlrg245, Magmatic, trapman, sergio_ruelas1, caroduf82, blueskycrf, Some Guy, gunjan.bhattarai, scharpentier, scash, cathaybrent, wayyne, brendanrmills, fangu42, wiillem, cheeka, gugish111, namchokdef, muenzer, Reklaw1973, aurora999, Monarch, hcvalentio, yamanq, sorgd, varjo1, StevenKell, emilat, JamesB, LordKunrath, sahndie, dein_kommandant, iamchriskelley, epicfalcon15, Kafi, SherDG, Klondike, radigan15, whatnot, BrosefStalin, anto9uspius, GMark, Alipony, citizenkane883, Corozon, Judric, mrfinntastic, Dalcus, georgethebeare, BlueBotanist, aviator56638, antimatter, umbrellaPrince, Benjamin_Li, deadifieded, Computerion, -alina-, sealyt, Ex, Kelyank, domdomdeo, Zboubine, vinniec7897, delaneyyyw, Histerion, viperious, eec221b, ggjeaneude, matthewtyler1, rna_enthusiast, Rivalium, jellska, jagodzinskaj, TB killer, B-Rabbit, potatoman12345, BichirM, UnkDevE, hwilkins, doodeoo, BKC, felix1998, antonchristoff, 18hhochstedler, pszym3, saidai-no, pansap99, Acuzik, kelvin.yu4, Crolius, Theknight7, RhysMac, Nightwarex, Morula, dominatingX, Vick, PVequalsNRT, televisaos, simonvozar, phantomrequiem, mrcookies, moleculadesigner, denisb, athakore, Viktor.mg, gpc5000, Go Lem, boomdude63, stephenb4, blaverentz, Raju, zhenning, haupt, StarScience, andrewcailliet, hualiama, scobyke, Ruda999, Z-Man, Unkn0wn376, kingboo, jedimasterhanuman, atomoton, hamtruon, Kremmen, Spooneres, Hypernova, DarrenLott, bolgir, tdarron, polly66017, Lalourche, yhomas, tjxp, ajvenaje_md, Kamalini456, dennisprokofiev, pierceh2, JiggidySwiggidyGiggidyRiggidyJess, Macjack, blakeballinger, fra888, Talmage, drake178, devilish, mjythgr, JohnCena, sbourdeau1994@hotmail.com, rye.coombs, AdaptiveCoffee, greendude, Pirus, jmayberry, DnL, Towel42, pinecat, zacharykent, timotk12, CAAndrew, Inosen, Fawn, pensive_sloth, ezbot, JohnDangle, artarius1, solar_traveller, dkbarnes2, emerilin, james.mackay, ethan_13, diamondscar, drakereborn, Handerson97, Ribonucleic Acid Junkie, MegaManSam, slymmer, xxxanderrr, kchambliss, Zainab Badejo, fafnir, gzhu22, kvalder1, soupnix, animal13, Dexar, san7647@gmail.com, aet36, jaycebradley, salmykanit, Delta168, jzaki1, hell2950, inchoatewaffle, Hank McCoy, EdK, Ayedyn, MCuis, NickyNewark, Strikefusion, natalieweissman, Cactus Jack, saxyness, Wcarta, Garod, LeCHIrurg, DeepRiver, xadad, Source de Lune, StajaStorm, durka1000, Moon Man, Kemono, argo007, jAEROd, markshvili, electro2678, JMZ, LordSoulis, tartule, Mr. Solo, shridhar, reginn, Nilbonur, mynameisbob, vogtmich, MrMean706, hognmeister, hkhan249, SumanV, pebbles, psifio87, tajclan, eiti13, doomie, yayforfood, LaraGazetta, Matt Indykiewicz, dskkdskdsk@gmail.com, Broomster, TrulyAcerbic, komjum6, BurtonReed, a user of this program, propheteer, goerch, spiffykavu, Dywyn, DanielMaxwell, RaeVigil, Rzv314, yong syuan, joey70013, Wolf_01, Mikaeru, weaverl3, Moon, laurentbaba, Leila204, broofboob, Crat3s, zandramssystem, mtor2, babyFolder, migs, Duban, sasevy, tate, Jumanji, Zakki, EdouardBonnet, AaT, migulrocks, cryptc, fcell, clementin, syrthael, JMStiffler, alhariri, starkwinter, Loneliest, veer, tohtorisykerˆ, MatthewDrucker, rayhead, starski1490, Anteprefix, Nazamroth, sc, kevinyhong, anthonyperal36, jarrux, kolaps4, xb, ZeroKelvins, tardigradedg, Sparrow3183, curiositycreature , M-Stak, Safler, ottapav, ryisnelly, thanhngathinguyen, dheitmann, Morcha, Timm6539, andreyz4k, cave felem, SHv2, pez_eterna, MicroW, toneranger, wolfeac, azsr, chw68, thatguywithhippyhair, Salmj, nate300and4, wyom, telysea, johnsonalyssa325, AbyssalCeph, a52, Bio4Bio, LadyEnoati, Jo„o Bernardo, schmuckerlucky5, b_ccruz34, RisingNucleotide, thserp, cd73, sixnorth, SirriuS, awangii, NikkiNeko2012, McHunsdonstein, slaterj2, artarius, mooper123, 02100, MartianTGI, brokenbadguy, jamjar, EwaD, alchen, upquark1, Josh198t4, comorbid19, milnus, Botvid123321, VoraxRaptor, ahearnc1, ahsan8244, Entropicana, Quantum, Aleboo1, miii_kiii, Spenni, IgnatzKackebart, SLSkiwi, flotterHecht, fayddle, Gage Boyd, johtso, Saitama, AlexMercer, Spartan0534, drewman, Doctara, cherry39, wingfly_1234, LFP6, CheatTheReaper, berserks, cavejohnson44, Bill Nye the Science Guy, koprocesor, Deafeater_PRO, Trip, hpets, 4plj4k, Gotoza, VirgieP3, user256, Gennady Stolyarov II, quest, kappa123, evanders, pearlofdarkness, ECAP, iwayzen, Infinity40, 0ah064d, Tauchsieder, Deadport, filipT22, alrianne, stelios3, AlxrrY, yaldojai@uwo.ca, mdwoman, IVIadScientist, Frid.be, alynora, btbam01, greatdreamer, Zezel63, kartonrealista, luisraposo, spiritofbodiejulian, Ishimaru, ilovepi, kaNino, Janimbus, arma95, liligirl1234567890, sarkonic, BrittGay3, gross23, wolfhorse13, col_kurz, amaniduncker, fifoumax, abbifede89, bugspc, TylerMilam33, lamartinium, rsardone, Ulithariad, apocolypse45, ColeV, gigamech, SilentD1, abuyagop, dusnikj, bicykiel, caillou, Mason2222, cacamach, Thea, mscp, JulianZhu, jy3, MitchSqwuu, Tellis, thezymurgist, Elposze, emeraldflm, veerkhanna, Consilience, ViolettRein, thebigdoodoo, yacopoc, nkarray, Alejandra91, RosalineM, wolfxana, tamc, vatokykaika, nomad72, Bananadine, infeRNAl, enzo732, ahsanaamir15@gmail.com, chuckleNchu, Madame GrËs, lorolo, Chanish, pagejade, Catcow, dragonfire1, Floris.s, noahsterdomas, bluenightcry, uhrag, metcys, nyegy, NVxWILDCATx12, ethong, frolickingspud, collegebookworm, ooloughlin3, catholicon, ascience4, Onymbit, BradenT77, annaofwonderland, KIM ji yeon, Richousrick, StuckinmyHead, alwin, mrjes, branzau, slimjimmy24, IZmanetje, IlPero, DylanH6, k14, Chloe_Kb, dronkihot, baileybalouch, Beauregard, Scientist formerly known as Rick, springer.chris@gmail.com, Anteers, starwars11, aakspuicomterixx, thedoctorgrace, Kesandro, Dikiyoba, trebor8201, it1023, codygeary, StAr, Bapt334, afoley947, Emeraldguy, Catch-22, Cdixson, kycklingar, gurumi, Elegans, Palatura, darwinsdog, jonnyflash, Firemyst, Daddy, Muzzhum, xSkyHigh12, SirFredrick231, Natabu, s202805, murphyhc, drewbert, Martian720, antsmarching, biogrl, White, GamerOnov, m4812, ammcelro, antreal, benacte, PJ, JanTheConqueror, NoDakTime, graph, kinsir78, llamadog007, nathanielbuck, Jpatino, kmccormick21, JEPR83, unlocked, deathbyfunz, jdude104, Prothon, rglowrey, i_am_syd_barrett, Seanser, BioChem1996, SeanMurphy, talwara, rtdub, blikdak, clayjmck, kovo_05, danlan, doradears, rsingiser, L.Klein, Aubade00, acipi9, hobbitluck, unusualbob, Nikenik, mohammadsdtmnd, mreyes, FlagFlayer, aubreyy96, rlharrison, Theocrates, lucaswinningham, salamalekos, sggursky, Freddog, gdnskye, eshun, Daderic, zoonut, EternaRNA2016612, ClemDavies, Artdog2005, Benzyl, karanlyons, maraam, heard-snow.m, Craahcourser, Leontis, Gummiworm, tgusta, valeriamin, ghosth24, WelpeAuto, epicFaces1130, vfrankenstein, matthias1373, dzsainz, voonkit, mitchowski, MistMask, mws, SebastianK, gwd1093, batochaussette314, xenon54xenon54, TheFoldinIowan, dwie, brapiro, mboozer, Sunder, jackmyth, dStreamline, mmariset, Spaceninja, CJH4, amaragold, Seikre, BrendyBoi, mevenvirgil, LonleyGhost, Sova001, cyanhivgp120, fildm, noahwhiteman24, enhandy, BLT, Ekiastheboss, trio, gem, amenephus, Gnida, kareybh, Anon1001, stevenglasford, ZenNOA, unseenantidote, 4terie, santiagoneym, Knight2050, wietze, Hydrvs, Tiro, a1b2, avery3r, Dischord, tuete444, mariamnd, ggrafham, marlasinger1470, Rheannon, speckle, titonus, DrCooper, nan, BroaderKey, 20043@wdstudents.com, ahocevar16, Niski, rrorapaugh, XXVI, starmile, 2thug, solrac, pcbPAK, Zavarin, Sirocco, emiliomendezpaz, 19772412, EHzg_Johann, golddragon21, Thep, Vergalon, eszipf, bekilewis, kworzy, norkie, siroderab, BetterCallSol, Zertu, TheFableFang, applesplice, DoppelBia, Saimon3, Cake or Death, mariska565, Lysergide, ghita, GamerGaming, Valdasgugu, Chadwig, steel112, Faithless, GeoPointer, poorigami, HarrisRG, MadRabbit, sherwoodtristyn, yarinetrobles, 2MG09, Kha'ri, Maristella, lapochita, medisad, briantingley, TerraForge, wadmanc, estebantucci, MCL, bialy3, Michaelaj1, kjtw64, tjscammell1, Poovent, Cpt_BA, nicholas938, Beligol, Noxbis, xxsodapopxx5, trystero, Thomas_T8, KCong13, Joshuahendrickson, xenorolla, ravenpuk, Tubbi, Evoluxman, Liber, Malafunkshun, Adorabel, aschachter, Bsmb1, JulianDelphinki, AlexSlimy, frostbite896, Abhinav Pujar, NicolasB_FR85, Z., dragonswordt, razorgrass, clementr, etIIenne, Huffsa, erenken20, Bjornfellhanded, portalbob340, coldwater, BigStinkyMoose, armerand, JustJoey, patbowne, mmmtstar, shaescarr, Buckeltulpe, fafv, bobthellama, tsaini, patandwerner, Madison P., mollycmcdonald, michaelf500, Ttimmeee, tankhunter, JamesRH, Bowtie, CarterCrowe, sheine, ya776, Rafa, Nigel12, mrperrine, leppinm, Mike_1978, fsp98, Mathias, julykus, eamcgill, wootmonster, Junko, noahyodur, Racounous, pbaidas, xenon53xenon53, Daiquiry, elm0119, Harrisramsi, gomaddy, Pyroon, ishipsomuch, bwright8813, Yanutopa, StinkyWizzleTooth, xTibor, Elward, FolarinOnifade, blasp, layognahor, DevWolf59, Treity, alcryst, vanlear, Symon Davis, hungryhermit, ThirstyFox, hds123, TheDarkLight, toniypedro, Rotifer, thejojobean, howaboutnoscott, HansLund, cataway, Konrad, webmax2010, KurtissG, Dannyboy, Tucoffin, KarBeyazd?r÷l¸m, coolsombrero, thyson, khermerker, YoloYellow123, HallamMcIntire, BOZY123, Neptune, LectorEl, gabortoro, Derecus, hacki81, dheft14, machoota, BrunoTC, AnastGrig, jim2707, Lanrhartan, mkafzali, Quiztopher, SaxÈn, acentanni, Unlucky, gregor_patof, Jesper7, chapeau, JelliBean312, GAZman78, Willed, ATPro, aelahi95, latana, bo6, linio, SciTest, iamawesome, felipe.ellena, DavidKMurphy, jandew, Ralfee, kitty117, Kaavya98, jpatton5, soonspider, Bl4sc4r85264, LBlaster, Archaea2567, quiz, Theargonant, Gwidge, Kormie, osmarsm, Wojciech [PL], rachelriley, Ignis, Julio Jones, ich_net_du, kourama, BenPyton, josefine.folke, Anid, ChickenOverlord, leonamafram, celsobp, dtribu, Erik Samtmann, ZEENOEMESTRES, AdamantOracle, Max.1023, Onik, jasonprice , somethingcyptic, jules21091, joannaohoh, AwesoemLauren, Celeria, Foster, Krell, rocketdock02, Osuka, Angelflower0, Shika Books, kyle s, jentchai, poiuwert, mpajovin, puzzlewhiz, HudoGriz, Zebesian, Chaospro, andrei_wildman, Sherwood, jblaqu1, ebelaid, Joshashell, ktkat218, lisamad, Abby2012, colacadstink, odmir, GatoPimp„o, pksvsengc, gbatama, Heramb Reddy , AlienC4, Cobby, Comrade LabTech, AI_White, gatherer, Abody, default917, mitjakocevar, d7u8d9e, Nergetic, MeTaNoV, Crotalus, Algotastic, Corranmac, dhinostroza, lightningelijah, Oreliel, kariaa, USCmwh, xcube, mlab81, Edsterman, Tunkasina, Rechenkraft.net, Szopen321, Tiphaine99, SrikuTheNerd, remerald, TheGinjaNinja, loomismeister, leo2, Messer369, Tiitu, Oukikoya, Tygerbunn, lherout, 1158499764, Aces 47, niashwin, harryk21, sciencegirl, Ijangk, Koboshi, woodie23d, fluffy3, Cmastin3, joeHope, hunter4928, mraider94, jmfish97, gatsby169, bonyonggu, LouisJacques, Nickbot606, starryjess, CreditOnion, tobi123, Simmo, CeraTheBird, jlentin0506, AlexR, baekhyun, mspsychorage, Midnyt, kafshari, Naoki, andreasstinks57, elpimpon, QueenDianna, Tyeor, Clindsey, postwage, ETERNALVLAD, sselion, martynez, BiancaLCastro, Amushta, vincentvegaa_, aria, Nol, dsands, mathcheque, ItsPiet, SLENDER RISING, LRpaul145, Oineh, adakadabra, Rudi Metzger-Wang, shaio, sayanbasu, Applecrap, mdodge, britsie_1, slytherholic, brainiacbabe99, highchemist, TR1406, motto53, Jac0_15, Mei li li, dGameBoy101b, PigletKid, jansonsrolands, deblagoth, CausticWren, m9898, Abatos, LimRim, Obscil, legohobbit2002, Dakirnz, ylu20030301, satrum, Omnicide, ejkellerman, gjneumann, Nephyst, uihcv, LauraMars, layerofgauze, pnhoffma, bchrysto, hangth, nickswan, wattsofbacon, jgreen77, mackinthehouse, ExtraKryspi, ndootson, AndrewBlunt, bootmii, brooke.breeding, spv54, Cerox173, euan.chalmers, atanimal, Pacificly, supertrol, Yuta10, J.ham21, Timus846, Beniona, EvilFish89, Heek, Hippopotamus, lleang, Zbyk, apexpredator952, Rojojo, DingoDongler, suitkees, reko, Kenriq, sfrail, wize, Spiny, Torin, LeavingGoose046, slegge4, cosettez, Yacuzi, PlaidPilot, itarannu, DarkSir, HawksinE, mohamedamgadyou@gmail.com, jlin0519, JQC1029384756, han.brzezinska, Brenda Geisse, doctorkasic, jzhao293, Kottosix, tolup, mishel, regi_the_veggie, nmengist, RemyD, B3224, DRNA?, Peter L Barnes, MJAldrete, Naqster, YoyoAarno, sdjili, DEdwards6, Tumis, salmohsen, robertodelago, blakjack, Marcurah, tank0221, Yeti, NervousHuman, Ragdala, steelbear, MrsMeeseeks, Sophieroo8, hchan, RichardMckanobb, virusvirus, MDokoupil, arthurm, clairebradley, soloi, APlayerOfGames, mwong537, mini1471, Miner9099, SG1966, elisatroester, cub4libr3, herenyon, emothershaw, m2oos, elstinger, krailon, achui7, RoninAsturias, syuan46, Executr, schalifoux, Piotr_Rywciu, Pandafighter, anan1003, sandman31415, s3tspy, altf3, biocycle, XTHANEX, oliviacho, Synthboy, kanayamaryam, bboylston, magistra, jsanhueza, WhovianGamer, Xander, jsantiago, ealohr, jomau, RNAribobitch, gregorymbrooks95, Zectron, snargleclef, bruhitstina, Jumjum, firegod954, selfishjelly, petrih, samster91294, richy3454, TerrifyingTaco13, kingkeem, musicmanley, Boslof, pidef, sfrye, whenham, anthonymur, jonathanA, Alberta6808, enorm, Remilia, Chemark, lime, momored, MarkusBergmann, mariocobo96, elpeee, Awesome Quest, MissGarbs, MrGecko, achant, darkhound, vickyrox2, givello, ScienceGod, giovig, Patty4444, MrFisher88, romanus, xaythor, jduber765, Neil1234, TheKing17, Glitter2, katalina2002, KingCodex, Brandonlam, blakesilver, gsing24, Wind2048, Miniburf, Digighost, virusdesctructor, dulya, Grallen2016, Spihc, tazmanianshedevil, TiggerLink, Zagreus, mctanker101, amberna, jafennelly, BuckledToe, mmxrocks, Crit, annaml, gravebone89, dRedstoner, Querty, TaGab, Vinara, lambdahindiii, lucas98, GilMota, lordnaanor, carmio, ozdamare, Malakh, gamma26, Racrbobcat, priyalpatel, maximegogo, damigapa, 3Balance, RotundCat, carabear333, strider23, lalashh284, Seror, John Woher, qwesdfa, modran, MathieuAA, harder.c, JX518, nshauffer , nanolasso25, siddhant gokhale, morphatic, fixed_entropy, siainc, unitedmadcunt, TiffanyStrider, respawn132, kiri93, Daemonsword, Atrix, Foliper, jpereira, anderdyl000@bisdmail.net, PeAlc, TalonWing101, BelacF2001, mweitzel3, FelixIsaac, limenlumen, caranturiel, clollin, Bastian5, bingbong, Minigirl, Picses999, Ulys, M0X, ramulu, NTSX2O, Korosagt, Egavas, anachron, LockoBoomo, SamarthDesai, talasjudit, quihi, Dravd, kmares, rfm0905, Paternel, bt11262, heather.elliot, BunnyRabbit, crize, fromea, QVENTIN, PuffBall, TrueZodiac, Total, Cerox, blozeee, maxes21, jasperweyne, Fikoloko, fireasametaphor24, pie1337, Tikathalasa, Draco711, gauberti, Medk, jazzarazz, emily.banholzer, Yahuda, sataeeme, Denouchka, Lokiosus, 2020huntermerten, hmandujanoham, lassewe, mdurik01, drewbraham, aberry11235, imthescatguy, eternalbeast, chargyse, mcamp82, jfongemie, phobos, JimmyLy123, ShaheenSheeti, rsm080711, gtkrow, qwed117, archonxgames, LaCucarachaa, Adeife, esample, naylorm, Hex71, dragonlax, Stump Cutter, jdc, Instrumental, Myst_Nrg, cell(ebrity), sivatherium, GermanViking, DevonRolfe, stevasile, stevenw899, Bynmeister, Ashwill, Vyxz, Asho, modded master, allergz, AvalonKalika, Thomas Hake, kostyakozko, Jean-Bernard Tartalacitrouye, nlesmeis, ostorozh, eatRandy, Aberrant Drake, Mustek, Mr.Dale, dchen24, steelix14, Kuydef, greensun, BrandonMatthew, LHaines, SafinWasi, himateja, Pentrose, Lytala13, Supercafeine, JeremiahV, Thanemous, hallehai, jrau4, Jerdan2013, Lemetin, sachithm9, logan05050, MZA, azertyuiop, marmun, biogirl.lake, Filledagreat, geoffbrett, blin, Sigurokk, Kcfontaine, Futureman, garyhogan38, erland.sanborn, broger24, Coarse, Nukkunaana, kierstengodfrey, Crazy4Finger, Persephone, ItsYouGuysFault, DreamingCaliel, Eirin, Fernflower, Venderil, Minotoor, Luca_Giurato, 1SDAN, paulfox, Julia M. V. Warren, soulignighter, Headchange, Divra, Chintam, anthoni.noel, Caramac, BCool101, alewx9, astroblast7, ToninadaS, 212121, Deadbot1, Eterna Helper, qarlos, eladamri1, maddenh, alexwingate, Laura Justice, insinga5018, DirtyMilla, Marco St, thothdragonfly2, qtran22, jlspartz, nwu46, TheMoleKiller, rmeaney2, mharb, bgching, 83073, jxjosh937, wood89, dariki1, sonnyskies, hdong29, mfa, bsivajoh, CapralMandela, DettanKarmen, tdelehoy, arkad, coolninjarock, christopherdennett, crave4science, mathgeekchic, himmelmann, remingtontaylor, mathpathogen, asmit533, Jil4no, Elgryn, pvanwinkle14, l'ÈlËve bouche-pillon, jwhela7, oscarglo, Starflight, markmulligansr, Mippy, Baerdon, Extremist22, ottomaticman, stealthmachines, jpalme56, Patrick4444, ardavei, Nieljoven, nicer2435, roki.kos, Bernaden, Guillermon, element15, Kid_GameBoy91, zjosseline, smartspot2, bagelmanxx, Mairsil, yo_lass, masseli, iappelbe, Superd Sada, danielbelnap, laja4770, Drakorise, Azelef, hobo934, ScienceZoe, Anirudh231, radhikatyagi, osmunj2, sskala, ninabi, Tony.G, fluo912, evynbob13, eabrams3, nwg182, mdiggs, marsmission, memorykevin, SpartanJATA88, Dai-San, jujuBeans, Derick Hill, Judlas, cogitase, hnguy29, macclark52, Jenyah, xplocast1, Hufflepuff09, Stormcatcher, juhus, bubblecritter123, fuzzywuzzy2912, likegamess, jsong254, Leyti, E.A.R.F Health, Bethany2017, ssteph07, shotlolwin, chortman, Ladon, Akane_Akarui, kchung86, spowell22, Evian, Tchan, dsiem, greenshadow622, tharsade, ludojad, Kennysaurusrex, psandige, Archeopteryx, hakunamatata, Kronwerker, Feti, Remi13, bruno137, timlarrabee10, britfrog, rtkleong10, pbarbule, Uredian, awang263, ailuyomade, ylc193, Poisonfyre, izabellav, IceTalon48, Bankrott, TrenteR, himeji, AnkithReddy, tofox, AKown, slilienthal, abellin2, PDelly, gdrakes, sleekloon, LidCoco6, coolstar1611, KubaZgÛrski, laiserbeemz, shoeprich, JC2000, kenny2816, rlam73, xXrakista_nerd142Xx, aweitzel, NathanNguyen101, human004, littlestllama6, arantza1994, karniko, mattman571, tyster5000, xenon54, T30TW4WK1, jhill, davidivadavid, GnikˆllÊ, alayton4, sstaines, cicatrixes, hunterlock, David Dockrell, Ceazet, esiegel5, ahmedrassmy, Ferric.AU, ernewlan, Sharkkat, ewill19, mfourcand, Species5618, Arkantos, deltragon, Vinitski, Nintenkip64, Shaneeae, jcarlos6, lsjumbfan, dhdnstjr16, hwang584, PerryF, Margaret Dinon, dtep, gatoporonga, thesepaperplanes, jconrad, tonytony236, adimbleb, Ebenezer_Shark, gguido, bwondimu, gonea, Ultra_Lord, AchooBlessYou, eqmb, Fozilla, StaticalTech90, IMHELPING, Voila, grazzZapper, Recnalc, 17LuxtonFT, Mikeay, P-Criz, melindac14, draganfang, megansch, carpanellia, queq, zsf, lizzie1111, Grayn, aduncan1, joseph.cab, SathnaMoriarty, Felis_Anxietus, cozmen, clairehebert, Mike_w, alfrick, amckay5k, bakufun1, ingaffa, Weaslet, biomadness, coelhohunter, msalter4, nevillelevel, ruicabanita, L_Omar, bissa, Garnasha, SudoDave, rnahh, zwilson2, Polert, Smazz, JoveRNA, jmanlavore, aidenlkms, IAkumeI, space, Panoca, salcase, yanggirl1, fizzy9405, MisterAtom, ipo, Iyansa, Richard Hade, mrippen, sspeterson, N0inimeht, kho296, holyterror, pantushev.denis75, fffffgggg54, Domestic_chaos, roamerkill, YKTNSloth, Mennoknight55, gpspaceman, Starkyll3r, HiroProtagonist, crazyworm, kroma007, weak_chris, kruemelchen2, Adarsh, aresetar1, Anderps, TyrannosaurusRekt, Andyrooo32, sroffey2, hankpete, shshrey86, KHeyser27, Shraumanugvar, josephk545, FrederikVds, Freeboots, khamous2, Tudoman, Ness, Ethlaron, jedikidd, cuhlemann, xx , tristanmoon, zeranosdepozidox, Caeso_Lucilius, Cake_2101, aaron connors, aegnog, rakshaza, tmilano, plumcream, saimi, AuraSivan, DonavonEllis, Longdart, AverySebolt, jrikeman, natybob, faizkids, lokiu, woutvw, Smooty, emilyrush, WafflesPancakes, Pigoon, MrMeep420, rlblackbelt13, Uber Cheese, jkiestin, tvargo33, DrewWallace, Hollandalle97, mollekes, c3945080@trbvn.com, mliu334, AdriComb, habouhus, pythonql, ioob69, TNuWu, Donimo, nathan34950, Xeonicu, haggi, JFAN73, DeathByPrograms, Zint, odaliz44, MoleculeToad, kevin rose, telomar, biomeh, joen9833, wkcason, TheRedJuggler21, frootloops, tjohn26, bliep, Helix666, dowdyea, EX6578, FunSel, blanxfg, RoadSpike, pranasag, sarren, Borio, MDrucker, Jaunticxs, Mich4, FallenT, akhantha, C0wAssassin, XVCRT, TablesWillFlip, MAGNETA, leaveyou, Wafflesthecat, Maia4444, Shell, francesco215, jnorthe4, Sam Pettersson, Turtle1331, ecate, iswaffor, cchan827, j1, keeratsingh, Athanacia, Arthuriel, Minia, hohnd, The American Inventor, DIv35, daviddb, ferdsstyle, Jeeri, David_SK, Jollygreen907, OpenTBang, Don Lion, robertweigman, Tavikins, Max Tee, etexero, Whalet, hseitz24, Merivos, misterslim314, ericzuck21, riBOSSe, kcirone2, umpufnufguf, alpo, Geofly, Jfault, uddyn, tshall, shuan53, tmadge, rgxHost, vcarter0, Ccile, combatcello, timetuner, immuneinsanity, Toxicsneeze, Saotao, ross713, EnriqC, pblucas, karolina.pietrzak, hauterho, creator, PoppyScience, redstonator, ibatko, ZulaZoobRNA, bea_candy, dsizov, SimonWM, Korvin, unlucio, Aiden Pampo, Irthene, raipas, 6e7c43486c47723484e26b97f26dd7c6, lmabrey, OxygenFerret, mmcgil8, poochiethecat, Critter13, WiddlePug, nliv6178, ssengupta, seed_alive, strickerrei, 579365, hnilsson, flesh-pocket, jhagood_930, Erfevan, Wholesale, mot24, DERPSQUID, nsimon7, Eternamolecule, davidbai, dmclach, adamg, simonk052015, abriana03, furn05, bflaher, ETambor1, mekito, viettran1234567, Pokemongo, MiloMaeRory, .WolfWorld, a1rwalk3r, Noah Linning, hurinthalion, Rishabh925, kyle bu, jbowers188, Kunstbanane, Ispanico, bhgreenb, Nhali, fff, shays, agrow2, jlee2854, SLilienthal81, qnguyen, KatelynSomsamouth, 3than8or, Ardebar, CantStopHodoring, cas, ABeyer2, charles_a_rose, EldrichManilo, romanedevaux, Jashaw, Mcke, cwilli31, CreeperGaming089, ChillBIll, pcollins24, hellome1, katelyn.mose, bruno, 4science , fleshcrafterAngel, XeonTesla, esun7, tilbert, paige.roberts96, Algorythmis, syang, isaacdavid, FreeMars82, Picten, rezten, Trinity_, Krejberg, Flygunn, mbarsan, AxerHi, Zander, asilvag, MayMay, Gared, sliptup, enomised, Volodymyr, Danbi6, fghfghfgh, andre_cortez, SuperMerlin, yhper, SunkernSciences, Lhydelhart, andi_se, lderikx, foldingnoodle, cubedude47, danass, shobmann, snoddas, haynerg, Boara, BLUEraven29, adri0115, elad, sientist, Caramia, GinoTitan, Anseth, curranoi, dolteanu, bng123, ncase2, hvo12, Metalval, zylocks, karenzhao, pegasevil, laracomn, DAHW, zzd1992, Evolvedgamer, KimBurlington, BlackyAlex, ShannonChem1, dapbrown27, ArtFreak17, Alvengaan, Prokaryon, LauraMcMahon, Spiffy The Chicken, mmindich, ydayani, fiordes, kchoi8, gentelman_bear, maheshvi, heather.lee, Carole67, gabrielmm, dtk777, Bibriss, Patrick Cole, aTamSmasher, Curistel, RN, AlinaS2016, Migdalin, connorlink2005, bentmaniac, Spaceboy685, Cypherfox, rekorn, KrystineWang, LlamaLord, halocarter721, Thud, violetbrina, Postal, khobbs3, Ajarn Chris, Einstein, ellenofcrew, khaar, mangoninja, Deafwave, RespitorySystem, Dank, kalserules, cmazzi, crasher925, aadelstein, ty513814, ChristinaMetoikidou, thatfie, knj0610, ocelik, yzarwehbe, mbirds12, mintuk, zzjay, spovolny, Abena Adjei, OMJPC99, fireking16, evalos, Vancha, Demongate, jadeshi, lordcamz, Gerardo Jara, eevans29, anguye15, mariapproved, maihime, iyktyn, thealmand, Hiefel, ImpulsiveSpoon, mebious, slashghost, Lum4r, Jamie4563, Edginda, sbarret3, DOUBLE, Smachar, komo, QNVP, Purnima hariharan, noep, anvimar, GCLonginus, FlorianVO11, sllaporte, spmolloy99, awong234, CC333Junior, mliu366, GrandmaJulie, E.Blackadder, peter990011, marora8, MrX, mattia4496, AzathothTheGreat, purklecancer, d-wauters, elizabethallegretti, Xalndeer, ezhang26, belnoah, Nicetree, nvladici, XXBlankXX, BBH, tungakl, rpeet, jvmm18, PseudoLW, Xx_WildSmaug_xX, Joyce McCormick, hoxxymama , UnunoctiumAlpha, Geeter34291, SaninaVelee, Hemia, sirischuck, Omnish, ebergstr, LBFANAT, joantaveras, amberjp, Roads, TheBigFish13, MK38993, Tosatertopia.com, pnguyen23, profemoy, jobob, Cryogenic, toadyjon, Reli1218, Nikolai Is Awesome, egosum, novoid, AtmanHenrix, Dediles, k96as01, yhuan373, NathanGrimm005, momo12589, israel1234, flo, gratzasaurus, KBinauhan, Kerbalnaught, kamercani, ntgramstad, tarimo, fahme, M.Runions, Thebrosblack, flugga, nmhossain02, gnnop, johanna1904, kochieng, DouglasUppity, GooseBeef M.D., g5nty, Oogieboo, Jaggertay6, SoulWolf1227, Juliette, Sardinefinder, jackthebest, joshi.sumedh27, pmm142, ionut.axenie, Borrth, Rasend, tng92, katelena0, dcasey, nim579, stu_xnet, martindude03, sgilpin4, saksoft2, calebgeniesse, AKI_RAinicorn, lobotomo, TARDISflyer124, lmcfalls, GamerGEEK123, rocktvs, Britney Ton, Nathan_Kamm, JJREEVE, KeithoBurrito, hannahgrace1018, dthong4, Katokoda, BangoSkank, blazm, irnmonkey, Stranger Danger, zordonmlw7, Brough, Dr. Machinator, marbar, Tettecareca, starscape678, Archer2150, steinso, Bastian_5, sb12, craigc, anguye83, Miss Mint, vturnbu, svoorbe2, ^_^Anna, fionakennedy, Maximeus, engineerjosh, jray21, brookebenjamin, nsachde6, Mlg20coeur, tabu12000, kikiavicenna, cemkalyoncu, justincjohnson2, ObsidianCrow, conraeli000, w1ll1b0b, dannyderito, nycari, LiamCampone, mds8575, venemab, kwade4, accessnet, braidenphilpot, odrae, potatosalad, kidsister4, FarmerJoe, Nobody21, thopwood, GrannyHQ, green_panther, Zashiy, Jion, allenmalo, kalia_kona14, matrtin , ctobita, rusty2hip292, G.shelain, ondradoksy, naimahanderson, achen325, greenjac005, Pisobiech, kalleina, Dt615, borderwulf, sookie0825, ivybut, Storm_Trooper, banglesrok, pkriens, Razer, mchopcia, Mightily Oats, TylerFowler, gayounggu, xwang985, fabrioche, Madrawn, ShovelMeister, kint, jchingjo, rizabilgin, djdewott, astonian, pdeioann, Lawrichai, Stevenador1, Fractalus, PedroMGomez, hamster king, dsteph25, aherbas1, snazara3, azuredragon, thojbaek, hinomi, soha, Nathan Graves, ghty195, moonflower7, alex333sh, Cong_Nguyen, FifthDragon, Lab man, ehf467t, mlgmarkmlg, tsubaki, thussei@uwo.ca, Opticulus, ToriLee, maungj, leiak, hlefors, alanlam124, switters777, YuukiAira, squarelles, she_elf24, rgoyal, Hruzicka86, silverlava, Jacobbart98, mvw, alonsoaguayo, nihaowangdong, L. Lin , VictoriaP, aussie114, JaQuez A., BloomingShadows, Jozmo, CANIHABEAPIZZAPLZ, Vis3rion, mhrussel, abrow326, Wajeeh Chaudhry, oreroe, agreaves3, TrueTail, gparmar, ashrk95, fmushfiq, Shriya, dkim553, marcucho14, wldmn13, m.mcelroy, PyroMancer1357, piscesdan, Xiexion, AndyEteRNA, acusano, dkim533, innate twinnate, NoelRoche, Rigatona, RSO2ID, levibalint, BF killer, superduperman, GypsyFlip, smallvinky, Random-Weird-Person, Otman, KokiriAle, jun1126, Kuhno1980, Thegamingsushi, sfstika, donny123, Overgrown Lizard, thomagra000, cerd, kodra, MrFisher, ewohlwend, xtox, SteelCastle, akhalil, baylw, Kirbypowered, Pineapple2Face, dimozg, Haku Temaki, Kang, pvanhook208, atributz, Silmaril, kizmit53, annikac5, blueivy1117, Xephum, bentlogix, Maximus550, mihnealook, EmilioSevilla, Caroline2334, ≈rgangsdreng, @Scientits, S1961866076, Cucumbergirl, Daholsapple, epl500, adouglas, knighter1, soybean1990, Jym, kbozek, sarelg, neonspacecat, JeBuzz, nicamarie, sshans, audio.01, mcapobia, jblaqu12, Mt. Vasuvius, ralichte, SlightlySly, Matthew Kuan, Crazylady, genops, aabdal2, SalCoombs, thefallenone2204, afranc9, skaterkfbs, jegarfor, aalassir1, Tigerlionwolf, diroku, echig, genetech11, alang49, AlifA, orangeninja27, zork2004, alex02144, salex, ashleybielski143@gmail.com, TrackAttack, Justine Staley, sdelm78, MWeber, bdk, Cathrin Lionhearth, Gondar, pumpkinpi, Almanorek, chefshawn78, ForzaCar5000, Nga iti, deadlypliers, magesparrowhawk, Askapa, whiteflare, mpesce, Rowmanow, mcgrawm, ouv, Farming23srx, Gilchrip, billiscool, ryanhsnell, wxy21, scottgrant92, jdemse1, zareef2003, Sturmrufer, zeloui, kittey, mayanightstar, moleculaire, pogi23, mghaderi, niccanney, snath8, elliezack, WFoley, Twisted, Oogama, aadeoye, freedomforfreedom, Haojerr, JanH, slazarte, tuxmain, rutzph, aisoize, rnbetts, Undrscore, edwardtan, Alex_Logan, khulanu, esavier, Fyrebright, la .lojbanana., Obstmonster, NotaSF, Chronon, fish97, skim975, isiloron, jethrowu27, alahmadi, Jaepheth, DanqMemz, CrissZ, gameplayer888, Iralix, kittykat1, Verbanderbog, krawson, MikeDD, smenon4, Plasmarocket, SeanMullett, stefan_nikola, farfromunique, poundsofnothing, RichardMark, Caeruleorum, tilen1, hessexpress, jmichelle, GMXMithil, e234, Miguelython, shackelfridge, DavidC21, paulneub, cpod22, AgNatishia, thuang92, veritas123, Oesophage, NerdCat, NikoKa, tim.h.kostolnaksy, Zomboy206, CVJ, doverlg, Smegenys, bekeep, aliersan, sgould, Floof, XD9lizzy, ScienceSuperhero57, nathannau, svantri, e.pitchford, lukefarns, mugetsu13, Deniz Mersinlioglu, dtsyplen, NDDbest, guybar133, lucygettinger, vizo, astarr8, eS5e, RolandMoriarty, BehNing99, Churmak, Joep 68, Final Warrior, mjychabaud22, msohail, Catecapp, jeanagustason, AkumaDono, banaan434, hetlock, lipki, MaitreNova, mbaggot, chippyweekes, Kotakiba, bsmith, tzohar, gabs999, TH3R3ALDANTE, Yliander, Nekkaria, Bethadose, navtron, nalkhish, mg1073, bnowak, BeRod93, joelthetroll, epond83, goodandy2, taylrd, sorr29, leor simcha, rikpat1, miuds22, mrok, IHelpYou, luckyshot68, WhimsicalDickensianOrphan, aliciacouto, Desolated_Dodo, yqin48, pmposey333, niniel, kalner123456, foxxy, k3lly_t0dd, Elcocov, girbobjack, brennaja97, 1ayeee.jr, haydutraduke, kolohe, cobonator, Colen Garoutte-Carson, salish99, Somerandomperson, willbeing, mpierc9, dnorwitz, bowlsmoke, xnitori, Martijn Klijn, luiggikf, bsba, Michaelhess17, Lord, julia_grross, missmmoonie, aoteabroa, Portaldominates, toscano22, JordanDave, Aden2805, BlerStar95, SirOcelot851, Doakungfu, SadCl0wn, n123, Chadowcat, osimakov, pleco24, Kenzo01, hackkpo, carliem, ChrJohWol, Jakv1, mr-kim, Xan422, john4563, hardy3, gratitude, RedBoober, 94fdrx7, xXjacobXx, hahmed, 01001101, Terra Master, Gadge, kolosova, muh42, Jakob.Gibson, Darius59, jrp317, adeprano, EFER, mantatail, kazafox, michaelkay, sanagnos, togg41, Psychoripper, chrisceo, alisonpurcell, TheFlashIsAGirl, MÈsura, Viscorp, AprilOlaires, fder, jayfunk, whitmar, Jezzaboo, Ubald, StrawberryWaffles, banuorala, jerry.mi, broadwer, kkaminsk, pdmock, justmeemily, artemka100, saversa, Binusuairos, 602, Spiritgirl, nforesti, curtipau000, pfudor, kluball, QuantumShaw3, aliu223, silerka, nawaalakhwand, aru54, Gabs_the_lil_protein, dan german, bmusselw, AndyJay, Jwb52z, raph.desage, badcorvus, tata1993, kover00, mlee688, aboubou, cleveron, Xydron, 113568343, Akkaneko, Heysun, khendarg, hielox, arelyquezada, mochi, eefremov, bgburke, WEenYThePOoh, bmosley00, raspberry, BytesAndCoffee, Crimson.Rose, Nbukhshtaber, addewyn, jrosati, Glenn T Jakobsen, Onk@lo, JD186, sjcook, Commander, Tanakakuna, adefelice, minabenc, 15boolah, SlayerIssac, Echecs37, TheGenieOfTruth, amelia kracinovich, tonemas, shong253, Ps1, jackgbg, iforgotitalready, Nikhil, chronicAnimator, jogundipe91, ejoe90, eblick, keithtothemax1, woodytw, jazoody, Spicypaper, mspeight1, master_son, OmaroA7X, ncastaldo2, Adam Grieves, ThePetarda123, Tannet27, FireplaceScience, gotauber, notJames1001, Mattkew, dager, MarioThomas, atran94, isabel11, pbrazzthemighty, bbridge4, forkihn15, smir26, Trotski, faditarek, sylviakinoshita, Nick_Linux, sharkguru, Theoneandonly1040, ghlee1219, KatrinBlade, Talifmo, Tyberio, rachel.phan, rickatello, isporrih, kurumiheartt12, agm783, Azor_Axestrike, danimal527, TurdusMerula, Halu, RamonH17, Khaysis, Stem, Grolsh le laid, rnaklask, nikzaq, Hugoale, stancmas000, ncastro, Ornjpeel, Ruben24, nono_you_dont, sakul360, wernergouws, mimeep, ceta, crayolalola, IVT, ashleighhopkins87, gooper20, Mojok, BulletNick, drklimow, lpartida490, danascullz, ecowan9, Young_Ramenz, ecd1192, ami, clemecar001, anggasendiputra, firedrake969, noahshawnpower, ellieg, Generaltheo, IsadoraGenevieve, madakelij, saustintaylor, julien-elodie, kljs1972, martieth001, Capnkrunch, ragingasian36, bmj1f15, Hourly Kind, SEBOMBTIAN, JKOtterson, Alejo87, Shiser, c.campbell22, Ilovepie, adamw, Mr.Smith, blah1878, doc jon, Hadeed, gent , DaMaster5002, ilost, HenryMartin, jyao1, kadir, Decken, GorpGorp, Eirok Burg, nyren, irresponsibleNinja, eternaman, greena81, cameronpalte, Valentiin, Ajgreen04, joseph a austin, Zeetepez, HAPPYNAPPY, LailaTheGamer , Mamadespik, pmock, maks.bond, WigglesMcMuffin, eoghainam, 21Martan, courtneygreen92, linwillis001, aubreymorrone, XxX_YABOISANZYFRASH_XxX, vazquez8, TKAK23, Susan Gaughan, Jovonndac, BrandonVan, Egodeus, Ysoscel, Yolonda, Biotox, ssakhi, whosvanessa, chunyi, eprescot, 7g3p, downinglibby, msmaclennan, flyout7, kougaro, arxidia, memgineer, Jsenecal, shamadac, Kingsteven, _147, adamdry, Nico Scordakis, alexjansink, element108hs, hukey2, alramirez1, silvargent, yarmin, kbwarlock, alexandreabeh, Camillou, pedanticaardvark, tiikerikani, aries713, Aj calle, klaverjans, Funster10123, jbuice, shb, vislous, ElliottB1, Taco, andreagarnicamunoz, natedawg49, mishfa, Faith Washington-Law, jkmcv, Devy, ayezur, sana528, davidsbond, cbrain, DorinaJ, HiPeopleMC, 0utbr3ak3r, Pianisssimo, Lenchic, carugo, a4368146, plmcr, MoonShadows, bazooka201, iriswave, tiredguy, kwh, 578780, julioflammenoire, theIOis, ListedMyths, ahysick, notyours, AstroPeep, bmillway, KimJongBill, Nick.Trovato, cybersun, Samantha_C222, moa168, Kyubito, GIBRO, Manarchist, kamisaki, ryanmcstylin, DionGames, skrent88, MChiusano, afsahafzal, Nessa0311, Osiriskiller, 22minnis22, voyager1, amathew, Kayla_Stevens_Schmidt, tumorraider, ccshnitz, mzhang5, trophe, Gracie.com, justabiostudent, rowaninja, BuildAnkor, queerferret, mcwasser, aagnes98, oman365, Juts, c_danovich, Evyslwyn, nick52a, JjUuLl, Jlim, VsAcesoVer, marcoleandro, TeresaFF, juzeddirna, ArtiSci, rybosine11, Dornito, casprus, felixsiu, B.vallat, dantepa01, edtecot, Megan333, zezice, Ribocop, Alianwari123, ABGriesbach, leoelbarto, linaha, Naomi Feldman, aloha.natalia, tberkness, laynaaf, FanderDander, fatimakameh14, lilfish, arlychay9712, Ionator, jordan12345, nasoboem, issathing, Augrey793, Unbeatomer, Kejchal, Crazyrice123, AbbyRagadio, pascalblue70, rapidfire, Goldrew_Amp, lucyjiao, Dawnsky-The-scientist, schmucker, kimwue, LagMasterSam, Apekatt, ScienceBroughtMeHere, HartySparty, cwanket, MontanaMuloku, Landshark, nofundamental, Cean, Angelica20, Alleycats45, jdmclure, 404random, Nike114, Duckman23, mi036, rider123, ragnork, Najimac, alina28V82, SLayGunner, BluntDaddy, Ibar, dianadockery24, scaintech, hiramwainwright10, BG89, shettler, Maxovitsj, belugabcool, Mattias Hermansson, rdales5, Joanora, Caduceus11, RiborigamiMaster, Msmrme, bdsoccer29, thedistrict, nbiley, DryToad, Ray0708, Calamity, emondal, Feardred, Leo_Gecko, breku91, M31, Kanta, Kyottee, nickyjo1999, desmond, lappaja, Harosn, Afterman, TheDefier, pioup, Zachattack525, lori_n, oem, zquoid, sarlamea, PG, doglover, juanjon, daniyak, yxia87, gecochran, ssantos4, Donutmassa, dave342, geek, kskalski, Vr?ina, HarshuEdu, Dmitryser, graceelizabeth, rot-proof, anderson207133, Error_707, dthielke, Shad-Knight, Sullivano, Achieron, Kayla S, zbjes, Neo201, TacoDwarf, Tyromaru, whitman5, MadScience, ingolemo, yutuyt, CloudCompany, blaize2377, jeesanchezdo, xphamster, RadiantDreams, WayOfDro, Northmen27, Blamishs, simon1094, teseting, mtw687, raman, Spaceport, nhanhhf, shahrzad, lbahmany, seanickel007, RomanKantor, paola, OchoFlames_808, mahdiassari, darkangelx, User42, polluxxx, SammyLerma, 341464, Nerubicycle, Agnes_R, luckyclover5, Gumili, 12lucyx, bizcasobes, HenryTheNerd, 3030, clockworkthinktank, linarie, aab70, lcbvianna, Batagang, ozzzzz, fedebio, samtuv, floodcam000, Lorathor, Wilkis98, allyrene808, Vade1234, Jonathan.Socoy, nateaross, ehollan3, mregnard, c_to_the_c_, regfish, biomadnezz, florashaman, Tiffanny, DubbyWubby123, marchisi, Becca Rose 2280, Jujuu33, Echuanga, Medicine_by_video_games, Feetzee, wchan123, aghosh12, skaplan1, Oesirpfy, rosie316, sof, heliotic, farkasosky1, Burcin, KrizzNA , VirtuousCat, Gonz042, zombooty, hanto, plat1n, L.Hoy, Aotearoa, friis120, Wojtek, Raymond G. Feilner, Jr., parichita, VDoc333, dougd03, skoff, ET Hazwire, Edison Amery-Zwartz, ThisIsFun, Aaron9101, Lis_atic, gt1234, pakstrax, Melony3, Katarzyna.Kwiecien, kreol, Leky, mcbride, Rhapsody, gsat, superethan, person_, lallllu, jhcolepiano@gmail.com, Sycamore, graenor, Noobix, hjohns29, leana_mindra, Duikplank, raitan, lililia, ggemme, kademing, bgw5064, et_et123, vincentkellyy, ChrisScientist, J_Starwind, KHSMA123, saraiispoppin, yasir, PRH, AbsoluteUnmei, nickr312, jnchappell21, machovec, Naputi_Today, Liloute, niv, stringtheory123, Rookiedu03, titousensei, DARE2, maxo, Smitty881, rowan42, litltex, Polling, jpark189, 0BlueBird, Plip, svillagomez, Eagle Bound, jjaffer4, FixTheFernBack1990, pab1990, cashew22, khouser4, byuan, johnbob, Georgia514, loyero, Bu?ka, emanuel213, Rance, cyberfox, Ekat23, blizzman1999, vladimirceman91, hedi, earthbound9, thefool25, Piggy, khanlamisa, Rebs, SethApex, Leoco, danielmcgee, JudyXu01, Belac, Loic2003, Boomshroom, bigredmadcity, EpicGigglez, Ketcham26, wotan8000, Snakehead38, Anjani, DAX5919, kackley5, tony149, Drunkenrhyno, Sarina, misa, Livogom, scienceisamazing, Yolo2012, JBogle, goobgo, cblum12, kpines, columbusinaction, criolongg, EeveePoke, alibobali, boltblast99, agrxkm, Greazmonkee, thematinator101, Helkost, zhto, nitsuje77, dinakha, corbineau, yxu442, imedeiros, may266, notelijahwoodsirl, rkart, mcardillo21, rsfmaquinaria, et246, luckypech, Sniper910, Lord_Aleptt, fydpfg, Wendig0, reyweld, sgochsmann, EagleEye22, christineortiz10, marhwini, Cupa, missivibleninja, noseinbook42, Funtagira, Addock, bunksteve, etherealducky, angosu, OrangeYoshi99, dicegamez, muser, ginaT, mcf, fionnan dunphy, eternaLwarrior9991, SheSoAlyson, orangefriedegg, islamgabi, defrajac, Skowyn, allisun, Caomai, Kgomo, ch25042, Shad, Whisker2005, hasard, Calliope, mustha, Leafsparkle, Cougy, draker585, Valdis, rupal totale, t0903591, lex555218, Macro_Lens, adroitPrognosticator, yanak, Lias Hosmer, cooba980, nelover8, ballin_with_daniella, Sebcarotte, shinamin123, NoÈ des Diversity Guys, jilly1075, lgellerson, Samworth, ssk, Lugs, mlowe, stepman5, tiedemies, franfrankim, Brennanscore, Shaolinchi, jiffy20c, jakemhyatt, MTaggard, sergiubreban, Link Shaoran, Ovorion, Fawkes, nusratnobi97, drgn8d, skeeler, akiraaaaa, Buttergott, Vegetableman, AUniqueUsername, The King Of Clash, weltonal, savanh, Tutur801, Anj100, j.barnard, Xellonox, nongenderedentity, dv_10044, jasontong, abehere, crystal chang, phynx, Martian_Boat, Nohic, Gamingscientist , eloipieux, Elite Player, geogeo21, mornplus, TeddyBeerSebas, cburkett, Zotshot, NelsonHMarinG, notdeadyetbob, awesome5008, StrawberryGS, origamer, davsalazar, antillon-beatriz, ant21, sophie.morois, elliottr, aliah.o_o, Lena E., Pedro Vieira, nodnarb775, adam.spiecker, DNA Master 007, svanduffelen, brooke9819, soadreptiles, kikidy, AlainFromEarth, armbreak, dtmclachlin, jp1687, atypical, o======8, mgupster, julianguyen30, Jojo2016, Haydo04, mcklsk, yfan84, Kumimasen, psychonaut, ventusvibrio, kosmasg, TheBIggestViralRNA, 09Q-SBN, jamiebosc, Proton, jeff0s, EteRNAllife, csziegler, Stefan C, ninja157, Johnathan, Jimjunlop, smoore96, wyh, AgentOfBolas, Habakka, ptolmey, GregLam, basseyabit, TroyW, Tribble_D, TheLeahpar, dimadan, mat20002000, Ditjedelta, Swinub, os10, Tunabun, shamrockp, Jubs, hreaper, Schmeckles, ihavenoideawhatimdoing, tiberius.duluman, AlThePlatypus, Xx.Wolf.xX, BIC123, adalel, Jovahkiin, kimberly.b, anton386, okawecki@uwo.ca, tnrlatt, Jesse Pinkman, baruch23, nil, phndl, andrew3511, ToasterBoxx, mchui, zarry, cayuni, Hammy350, Galath11, Bruluh, pvbookworm, gentsilver, dobster, Mgb0, Spug, CSteel, Hyenabait, Maxine, rhymeswithsnake, longtrang651, benpaduch, wonderben287, Asimov_The_Great, Marvel, nfried4, Wirvend, Immortalharp, andrei.ichim, ricemomma, ellianat88, t.asplund, Bilsh, gab21s, casare, kmfm2005, crazed4now, Jack Vera, seaslug84, Knoerdy, Curt_Folf, domah2, csfraley_rna, jacollier, daniel sun, abcdabcd, jwong521, bitman, Ine6, johnson.o_o, brandonta98, DarkAndromeda31, leian79, krismuasa, bjorncal, thesaladchalice, dakkaredsquirrel, autarch, matthew12taricani, klee667, yourguess, jsisidore, zellyana125, Bonojora, Fabuloso, Thunder_Bug, thead, enakas, ralphjin66, yaya671, ldorn1, Random Guy JCI, Sebaci, Lauren193, Kairos, gattler, Pumices, Grimwind, Zoynmkxq, otgw, sea7nc, millerm10, Neuo, elsyrin, lucelletanedo, idama, clone909, FinnZabaar, Frostterra, Hyva, bucketofsquid, crowan641, rbamhd, patszy, shavera, Max Goff, mariecorradi, breannamarks1167, sarahjihae, Pwoness, bonkks, ZyTr0n, strongbow, birdgenders, YeOldeShirt, 123928, jessfrancis, Gii2000, caitlyn.johnson, PishaMeans, enrA, kharyana, Mr. Snake, antosi, QIRC2LoW, jujogio, djproctor, noodlesnz, wstolyarov, Mehal, jonu_the_best, dennis16, sirrandom, sahmed18, hi529, bookluver132, lissisa2003, Bruh.its.rheigna, KylieDam1234, ptarecte, Eric IV, peepeetee, shawnxm03, mbohola, liaha, ztron99, JackDBass, yolao217, Post Script, polko14, thomnom, MikeHatfield, PrettyBored, kapkaart000, Cassie_C, esoryllom, Coppolino2002, osarker, TheWeebInTheBack, RNAdolphin, dtk999, chorus19, microbemania, Kreaturez1010, Stogsdill18, 19newcombb, breannan, jesscheng, etheenglishguy, SophieBooBob, Chelipotamus, nayfann, aurogar, kml, minjimlim, lixa9, Hannah Demers, HÈctorThor, lucaPasinato, Youlius, jcapp45, khiser, domvalc, john132, 312, Belarnon, jamie.bosc, Kaaktus, Sopheak, skrphilip, HyperLan, khynes, sbwtt, wow, goatboat, Belsinger, Mike_Eterna_Science, CriptoRio, Catt27011, pifpaf, Doadoi, Stanic, lgrainger, 784328, jpalmer44, ringachi, paytoncox23, BybyRONDIN, John_Benton, yokotopo, zvit, Vonrek, hackstarter117, hypatialovelace, sghosh1, RunRudyRun, brooklyn.kelly, dnanua, ncortiz, RonZimmern, PheleshiaPersaud, rakeshsahu, vtsongwriter09, skymagee, holdenhk, epbryant, cjmgames, mousegirl018, PÈpita, Onymbyte, profbdcohen, daanvanbeek, Navigator, lucien38, Trevorscow, WD3k, tojenanic, 1433, anakarina8, ViJay7019, yasminej18, jo.karou, HMK, lofty133, gurleen, mscollier, BaptisteTesson, CDR0420, Stalk3r, Matalos, hopalongsappy, xmoopie, Vini0101, randomblakman, bioismyfave, alisenchung, monkey133, basiqueballl, numpk, teresabg, jenzhang097, hanman69, miles1000, JuarezJac8, shorelines, 118lf, Farrout, Fealhach, mikkamikka, aconant, SilverKnight, ComfyBauer, Neythos, juditbestPl, Draksis, ilovepickles.com, ILIKEMEME, ampo92, seanzhao, caeleighxander, chelseajohnson90, rugology, jsosne, jorabanzai, tresus, ExtrementG, Ederporto, wraszowixu, malijah, bulletfire, pratt.williams, Jonel, luki99998, TheRedX, john paul, Ficik, GrassyInferno, jrogers4895, bsmall, Mizerable, Batpunk, masonsyd000, KirwanC, McRapperMcC, Wilfried Vanhees, Lhiebert , xxpaulob76, Aid11snake, CJohn76522, mufintime, BSLangley, awemanrank100, me2cool8, wingfly1234, shaimaa, Nomar, Durendal, dschwartz, jb1516, zobi, daleonar, iamgarethj, 2530289, Daisy_Of_Doom, someoldhobo101, ckirkpat, Alexavier179, Matthew.Nicholson, kilposhi, caiti-erin, Dr Pedro, pipio95, Sgt.Pepper, nizamra, Sherriff, zgordon2, wilson3529, brittstja, anonmouse, tydigame, mmetzger044, Edman, frazom, ketepi, Deirdre31, wasd19375, GigaDon, beachybum28, Leonitis123, donnadanyali, 2nd gear LUFFY, Wafflez007, Wintermute1992, nathaniel111, erina, emesse, zushakon, khallen, noahpinion, Geri, 1577, sohumd96, Stylish02, arempson, timlo, janel1810, Antoinedino, crunchytime, Juiblex, xiaoJu, Hatmic, Cr, Marvelously, sbadawi, omrot, IronDogo, cscott, Bugboy16, RishabVadvadgi, knonymous, LiL ickey, YrHopesAnDreams, jemrick1, Daedalus89, Tayloreolson, klovem, mvluong, puzzleman, EmilyWalters, chimptastic, 564391, gc717, Alonso, bivitarttus, rasmuskj, Trandel, RAVaughan, gregoire frere, cjelicah, idontkara, Anonyme, MABSR, Joanthyaga, jmaa01, ZacharyKZH, xenowarrior, julianguyen, boccitanus, scarysherry17, csarmiento, korille, killerlight, FaintingClown, YankeeDoodle, Darklordteddy, FRU, shadowsail, quietpetrichor, Anedlit, ThirdScenario, cheyenneconant, anmenqing, lwingo12, l2thompson, Brand2270, Dan danny, Dark, RenÈNewtonAlbert, xcombelle, stav, jneideffer, Jjamm54, Warpaint1263, silverust, tyler2002, werebear, Noshahaw, LeoBattlerOfSins_X84, vijyadrit, IrishRNAmaker1, edgar21, Seasonhold, Firedragon1408, RetroGameChan, lorin, Matt_Griffin, L0gixIII, DR.LCI, Kianna Mayfield, Namoon11, caverdema, alphadragon, ethanv, Veretion, JasonL, Linaste, lchamberlain3, graou76, Sophie16169, CoreClock, simplychrista, emmaline, gnclaus, winner, Desap, vm, olixx12, cybersettler, prestonholley, TL1, JazzyJ, salesslie, msells, edgarm, cspoonts, gingerInferno, alphameg, rosettejaneubalde, elB89, york84109, marychapped, eKTHA, griffinmic, duxhell, kcow, ALargeClam, PyroScientist9, enirei, Btgoal342, Elenah481@att.net, Ademir, enframing, Geobug, Onionne, Arjaeniodis, alana39, karlamichelle07, nba, AldonAshling, jaminrol, arminor5, LS1222, ashok, Siclone, Thej Bandi, NebulaM26, CEAHarfield, WajKnight, jujuliter, soundu, dmilsh, amktakha, garmili, Natachi, paigezellmer, gigglingCaduceus, shuenja, idara, suzim00n, Trapfether, dryasir77, Psiiborg, Papushoo, Alex12354, tim-timman, bwgregory, myfacehappens, somefish, shivin, Megan.Shellman, Ghost_792, TyTheSnek, vskvskvsk, Davie1031, Lui_Jime, Enderon, rhiju, gsim047, maximemoop, joshswimmer, BOO BOO KEYS, 3DCat, derederevo, MobileForce, properboredom, The Best, AnnaEThompson, Martiel, tigerreye, Bobaero, madistaub, RicardoLuis0, landastronaut, PR0SPER0, NuclearGopher, VanCleef, nnunoo, R-sham, sha28, Gargoyle987, merlins, a.millage.fox, karia, kjstrange, mbharrell1001, Tsumome, bray.sean, Marlyruelas, Kibeth, Helkin, Me2910, lafeedhiver, asterix25, ciechan93, volume, spetrov, royce, bullmar, shaaaaark, bcmonke, Ssschah, seanthoran, Jamescabangon, maxbu, Sgt_Soju, derajer, fangyu75, Moussa, lilyflan56, TopSLoth, Hannah6424, mstewart1001, rohithk1, Eli_zabeth, SilverSparrow, KubaW, jhpuzzler, ginnyy11, kaur, jgpfann, Thing201, TomorrowHero, YusufBP, si225, dshotz, MrSinusas, Guzz, jiwonkimkorea, DeadlyCreator60, Thiefington, GlebZmeykin, griffin21, bimmerville, baye.parrish, guigeek, GHMGOULART, hepster, FF!Sans/EF!Sans/UF!Sans, Bibibop84, aab02, thursdaytroy, stinkysonshine, AsuMagic, fippo913, Scrotie, 1nelsond, Diovion, Ghosttown21, Kevin Hao, bosspackers3, 118cc, Julia_Dickel, DarkxNight, asterisk88888888, turtlerock, Kirin, Scorpiodude64, drigerboy, meatballlady, KasiaSmyczek, nelly, verasue, yesmar, httpOmqCxpcake, hsquared, AlexKomar, TheOfficialJJG, 3rw4n, snapdaddy, Kaidjy, mercyfull_fate, ashwin.desai, Arcas, titip1995, juliannashine, MrEK, Anuket , zrosenth, brb2ty, shafezi, ndiazh, Riggs109, wombaticus, LordEnigma, jqiu1, aja4089, MCFIREBALL, nchowdh3, trondlax, leva7, NatalHydra53, judd.katie, Jaquelina, NicoCapsi, Gemlingur, nate2222, Nicolas Mulfetti, ethan.locke, rusalic, Collapsar77, karay, zartren, shadeymatt, CoralCobra, txukulun, munamohammed, KillashandraRee, the_lall, gypsymack, TheSouldrinker, tedy, superduper, ShahDarius, firstmatezombie, AnsonTC2006, sience9, eloyharris, Zukoawesomeness, sta1amin, nkatagir, 23TangRichard, lisastojanovski, vinbro26, SeaJayRNA, Joy_Fan_Roop01, cedric, a.yates4, david.biles, Sebie, gemini8, bartaner, carolynkim, Antoine ch, ertesh1, avaxu, Jared_Orion, kondratevakate, bmcwinn, Makala Nye, dprywitch, Daarwin, brutus12, arthuryeager, orklah, jone, Zenytram, thelall, JustGarry, sdabic, achouten, Frosfire, carrot, RNAMachine, jettripx, Helmut Coolmeier, vucko, amalis8402, BlackNight1697, Mooticus_Maximus, Dragland17, Rambe, thenning14, bio101, madcow123, ryand, Jfiep, giovanna_gracielle, gallareton, SarityClarity, shaym, Pako, Uniporpoise, book, nancyw, ghosteur, ARN-LLK, OffbeatCuriosity , TrueSpiritGirl, Jehu, SCarey, Aserita, mds_00, todd6485577, merinarasauce, olyviajefferson, nparker87, pablospalletta, chloe_hess, skrimon, FnnyRssl, Fierlantijn, jup1, frkmaster, Jordok, YariValtean, YellowGamer2004, 11killer17, Michdkr, drjevsky, pinkishdoplhin, daniell411, dshariq, 8wmasreas, CaptainZap, coderah, xarpus, ewalk1, TheInsaneGod, MENTALIST, DeniseB, kitkatyum, Heliceras, earld, domforfreedom, arecka, tubaflub, e721420, rynomachine, droricu, Kolob123, Geminem, Adaukt, Sachera, rtroxell, trollhunt42, Rinske, kbourque21, majolka, Meistereder, nerdy creeper, loveRNA, ezio, angassmith, CatFREAK, FLAmbuRNE1, apang1, Uderzo, pamela.norton, jkm412, Melissa.Miller, Khuran, Ecilam, Kimberly Westervelt, ivanjr89, QuiquePA, Buggbyte, coltonq, Hemell, Aradros, Linda.L, Sfeshey, alessandromattina@gmail.com, arunduraig, nensi, thebigchief, Abbygh, ZacBarakzoy, sgerson, HybridWarrior777, b00kw0rm, Light140, radiationist, milla, Amber_95, v4r5x8, sandriix, britneyh, Prince_Tat, pastelbleuy, Jwaemsets, HoraCZ, theaudie, superc1212, Lazernoodle, PanikLIji, rere19, janetmaymiller, AndresM, yehos, Bshadow4, huitgh, emiilykim, smcalpine, an0ther, XLVIII, namo, lizhosanna, ablueninja26, Wgirl32, maijacole, diembe, JakeTheGreat, Been, DoctorWho14, Fishkiller79, shinamin0218, Koroku, UrAnIuM, CapsaÔcine, jmalcol1, Cody.Winkler, Reseal22, dhrg, eiryberry, mklowry, OctopusProteins, karlb, A.D.N, sciencelover, GreatBigPhony, 301sebas, ibib689, BZZZZZ-7, awkwardben, danhan, jessie-huang18, filman230, capu57, fixmancol, hmp10, ziplock77, Mechputer, oriramikad, fedor3000, mathers0606, brbrenneman, chemistry, Nosyeye, Billal, yoyoyoyo, Sockbat, test432, rayan, mkewing, tobi175, sheatynan, Asmath, luthe, Irtaza, 2bjackson, Abc123Ultimate, PuffballWarfare, DrBagelBites, hawkyle, Cat Legge, JustScience, blongey, dredstylet, coreywhitten, shutchi3, 10to21, Carlin, jamesgraham141, frolickingpotato, hibobyoyo, airb, tryst1n, Themannn, kollise, ddeeeffff, jdbollin, Amunre, abcd123, Epsilon, Leafheart, jephnar, Lorgelran, HannahHend, molding noodle, Ana Carina, pl, gredick, archie88, 18kmcgrath, tehstripe, amit233, Preofessor Goose, kurt5413, Triangulum, Im just a kid who's 4, JavierPozzi, traviswavr, Frank_, Adaixa, Jose Da La Garza, dnarel, jennifer.graves, El_tequito93, steven123505, sarecguo, annisa, slc577, MalenaLy03, UrhoP, avalet, Anand7, Valko, kalex, Carlosgrr, datgtaboss, nbernema, JoshuaBui23, SunMaeShine, Piaf, AMD9009, mic.sca, Illyreia, swolfe, WalleFails, Zacharilliac, ReluctantCynic, ghandolee, kortatu, alclsqjtm, reneethecyborg, NatTheNat, Skyesis, nfernetti, Kalarily, 1617674, BrianHo, mrhottoe, Meepers, edenb22, bc32065, rogral, KupaTrupa, pnd3m1k, pereiraj92, tjv, Pinacho, 118ge, 0something0, Othernaut, IceJumper, LizzityRose, houbar, beacandy, AlexanderSach, TsavoritePrince, lorraine.smith, adwoskin, 340atlantis, frankydp, ThisIsMyUsername, theanoxirouchaki, Noru, masdar1, shake_a_leg, Stimpneider, agomaa, seny nguy?n, tycase85, IsaacRNATeam, giulianova, golux, david0mckee, babyengstrom23, LoveLife, KimPossible, soreyabeltran, mskibniewska, lollitafermion, zombieful, Nanachronisme, tobiaszkois, amandaashtonbooth, lilioniana, Heathenist, davisaj5, andrewmcamp, Taylormhix, Grimalkin, wolfbela, Atiur, jore, sjs2609, Dr Moriarty, Pr.Xinel, rivana, dpeterson@thecornerstoneschool.org, bigmanskey, JulieJ, Gabicat, Mkay21, Doltboy, julianguyen2211, jk70, CHaosDragranzer, Kaizer, acookt, LubnaKhan, derfred990, mmille30, SirRainy, ZenH, conradlau19, Snr, daviddag, litewave27, ezlikespie, larry.allen, jaroslav, hattiemeigh, red.arthur2c2, mia.ferrell, patistarz, ryliejk, catarinasiopa, mali, SimulatedScholar, Tritonis, Luk3ling, meowman889, ChrisSkillo, benjben59, EfeGulener, Amedon, eli.apple, szulfiqar, rawanA, Riskyshot, mcampbell24, Kaylamv92, Andrewwu, jakabe, itynes, xmen43, Burba22, dekusk, saepharnh20, felipevergara, Artoria2e5, Roflkev, jschober, Leschu, annainoc, thersippos, shefalilathwal, zaklinao, liamth, lostRNA, Amidinbancroft, Quasar1015, savannah tucker, gtrachel, IceRunner, trystin, Wesley.Estep, Dr_Tobias, Brici121, volrk, FatherWeebles, KimiaN, nothanks, adarshap, IncineroarXD3, raugia, kpham, skeletonqueen, rekone, Wags28, mathiah, maritumi2010@yandex.ru, kathymore, GoatUnicorn, r__alistr, callmescott, Juliana, timbro, alayton430, Adamf1002, Gqflex, Baccaccio, Saens, Togator, a_kiger237, Keyse, ffwdq, Acer-Rhus, malagrond, Demi2722, jouyei, chimeforest, joeycut, bhredsox11, twstdelf, summ1else, Bulraki, nlautner, Hecaton, kkbsamurai, newrecycle, Coolman60542, DrBacon, NeuroPyrox, klsw, maderjm, Gideon.Isaac.Ong, serpentea, deathlikerabbit, Enden31, mikechn, cookiing, Addie.Simpson, BlueDogs, ER2108, matthapkidokarate, Jen36, mattmatts, toshipwreck, corqjffp130, j_prchal, dmshapiro, CheckYourPants, Pete198298, masterchiefek, yudha, Amlfsk10, Alikat8891, Hugh_Mungus85, mufflera10, samanthers, eterna_player, cozzers, egbrna, jay.stengele, Titouan, jnorden, antoutou217, gpgraziosi, suziesnoozy, arugner, hmkuntz, Damiansteele, dstorey2, Khamul2k16, Agoldgamer, nachi, hassaney, azbcd123, Maxi, devilord, brycerichardson1, DeanoxyKeckose, AniilaACMA, Emily75, AnnaWaczynska, s.davidson, henrycat, rex the jack, JaneTheBrain, riurs0331, Blackpopcorn, mattlscx, malekith13666, Tes_Tickle95, MisfitMagpie, evanwroberts, Potentia, chazz, labworker, bluesmoothie, phil_andering, NeodymiumMagnet, 118ma, sidramalik, NullCat, WhoRNA, catalyticwaste, pipis, pikcolle, dennator25, doudoulepoussin, manongirly10, eduvin, Da_Awesome, Ostiole, mario56865, mhh23, Shorty94, msioda, horacepang, MineMaster, yudfghe5y3sg, zazz96, yevtal, jordanfield111, lecaco, Tyger257, ANN, SpeCtra, Pikapie5, Mahboob, SoBreezy, popdopdap, carinatze, LAW1205, kj415j45, Edgar10711, lorddragon, Reilly1, studdles, prplmustad, krodericks1, bluemeeple, Gannis, mikeywally, tolvera, Ludwik, aleizter, tristandu03, sergioyamasaki, Chris_WPG, EvanSMCK, 9810173238, Dreadmaster, bord12354, MisterMikeReed, mathewdee, derek1906, boomer52, hondalove49, jaquelinegondim, paleo2014, geemili, 20267, rnjensen45, Toofy, zhanglulu, C- ZERO, neonnitelight, trystink, BioRocks, M_F, 1RNA@atime, Stebe0412, Pollyanna584, gabadors, Firewall, Madhp123, airplane45w3, Outis9, faielgila, vampirepony, Nathan1, eBAHN, lexivdh5, Snailutdza, blazergrazer, pizzaemoji, Feylin, stardark999, kat_was93, RedSkidy, Space dogs, DarthMenace42, dofusk, matt7188, axehem1, Jakehill, messed17, Nandix, KarenFA, taylorknauth, Frazone127, lkaylie_, NuttyBrannon, jl314, Marky1994, BobJones467, BiancaOCastro, yamatou, aweatherly, SaveTheWorld, rdrpenguin, CadarGabriel, valzalan, Numex106, RienCus, Scythe42, RedSkinn, tiberiom, Wakko, kyumin137, ducker531, duffdevlin, acrogers, Arrow360, toryyamsd, ChiaraBells2, Kayden99, ebolacure1, shimamakani, mateiradu88, sean4046, CCTYx, IvanZhang, chingyinwan123, peanuts45, lkmo2858, aude, aeppacher, nidwhal, NicoPanda17, jimmyrems, 376390, Tracisullivan, JonahAbrams, oliviaugarov, Hubbard.Jacob, tiger856, alowry555, Caiello, dungpham12002, Akozu, d3xbot, sanfoala, ARFR, randyvasquez0, jschmiege55, balavikramkt, Daycody1, iluvlife12, herrmaddog, jerryfizzsoda, Ivette, mecca417, maddiemoore2001, Grimmjaww, basepairworker1, christineortiz, Joel1, alroc7427, ukmjhngbfvds, Motag8tor, marysims, Sara-LittleFRU, kowman, ButtWeiners, squish5, jcadwell, dillon.moore, YangToinette, Kaljiz, mindfull, narcomancer, 940410575, HHHarvey97, Okawashi, Detovan, goku, aresathena, Bobonius, Tuskarr, issailius, bard10, Actually, blooderised25592, levimcc, Adrijaned, Martiniella, rochelletr, iuzziel, eselmon, Mr.Phreak, enshem, bremlin, Neutrino3141, Gingy, fsysy, Cyber500, Adebisi, braidweic, Gritz72, pinkdolphin12, OleNeuman, chase_rippedbody, eboiro, olerock, OllieB77, sathwika, sherryhe, jshanley, Kenneth1024, taylor_welniak, robertocf, Felix93300, LMJMeman, rasyidufa, backster, Lazy6787, webslinger, nlivings, criptus, CoreGamez, mhornbuckle13, sapiens, emmahorgan, rebsbert, the45thdegree, beaumontbass, amelie.tsh, bhuhn, YouAreEmpty, nyulascsongor, asteroidboy, tuadib, trams, maxwildcat, cebeci88, Alex Lee, beaver142, dylanchyde, DanielCS, skatra, kojawak, TechGeek16, Snaz, laurasligar, absopoodle, roscoeae, Antoniop, Pixalstorm, Stretch45, sandwy, Jlamps, aboersma, ewilli16, jtaylor0089, UN4, chido, jrm98, MCmcswagtastic69, lukgil, Thevoidguard, alisare, Julius.Wulf, sabrina_bard94, Darthlich, jdikec, DragonSlayer99, saree, 6and4, Lord Naanor, hboshra, Ibrahgj, OlaBackman, hjkki, lumb, wandofgamelon, kghd, kingofspain1234, svperez4, ovidiufelixb, errorlog, abkernow, bezago, amaStarr, awaite3, MrFiveDays, emilyh11, pandaplee, justice17, thepope1233, mlaskow, ~closethedoor~, jdwest, hyperblade, jacuelinenguyen, ForestMastr, awokral, Murdy, irishakes926, Mikeypants, mielcars7, a.wald13, Liszt2310, deathwalker1, raichichi, Clarissacordova, mgranski, HatimRF, ecausey, Yan Von Triskel, Unersame, david112358, leverly, TheCoreyGreen, Mitza_003, kinga.urbanek, nmrkaist, darkmoon, jaga, Mamo87654321, robbie, cc1122, Marius37, jherrrick, timothy r., elsandosgrande, luis_93, rose7326, romsofi, Queen_MyMy, thewrz, BatJJ, zacxo, dand, sefaroth3, el18839, DocGratis, laurelca, Nyabi, anthony4tner, Cataclysmicseraph, rachelbug24, lemc, detective6, A scientist JR, corbak53, gagarcia20, abbye.gernt, ifni, toast62515, pluto, renuchepuru, Fistandantilus, SirDylan, mwlee85, Daleron, mrnolanmorris, C_Aarup, olive12000, huntereddington, LuvesScience, MadScienceDude, haase103, homr, Slenderman60, carnaverone, sfellman, Salad, Deepayan_Sanyal, gauree.srini, azerty, iijesse, kiavic, Pengilla13, Alx, gabriel.thomas, Dilldog, Bhavesh123, Noel, joanneklaas, Latrok, shelby.ward, edoved, Solipsis, galen444, dumana07116, billettkp, jeemusik, zork2006, Zacheryery, Jakarrie, wildfried, mathwhiz5, Ashfulness, Hristo, Zelyn, markthaines, jerseycitybio, noelnova2, caleb.coine, perezed10, srtokes, Dumpling_Trash, cutiebearjames, Ischenk1, cactus-lord666, gmontanary1, theinric, GermoSG, theguywhoeatspie, bonniejiang, akwaugo, foldboy, NeuroLiza, Guioma, cyna, brandonchou1, ssss4 , Laminariy, Brook, roman.kishchenko, AmandaCookies, pazzze, SweetReverie, DyingGiraffe, jgp, emoell, PerMortensen, elcapitan, Grandfather, VanessaCruise3, dmayer1, Austinquarles, TrueCuriosity, Burnaboy, lindabennett, Josh.Upchurch, MadSc13ntist, dareng, truman, haager20, emi025, WindRider004, gummi059, beck_ike_, Azen, anurashrestha, arthur2500, e666666, Raitmeri, salmonsalamander, elijah, traforch, peepeevs, Person5000, rgutierr, Zillamon, fotamas, coup1393, aundal, StasGtas, nightcore, Manesero, 9ar7e4, Ermagettin, apolloheo, NoahKlee, superkiller, alexonaci, hTRpardy2015, kamalparvez, iamgoat, ahonan, jYurko@stmaryslg.org, michelinseal, VALLPA0420, rahul.anand.1, Nemesis18, lampfishlish, Ill_Neglect, Frankentree, eva12, JustinPheonix, isaac_verdow, jumpshift, Tonner, Lalettayin, alicemiller, ZainKeegan2003, syn hd, ProfM, RVSdoctor, Pirocat1, Artarm, Blackish314, Spielberg, Biologiste Sceptique, wither, ethanoquenda, Nathaniel., The Floppy, Polika, pGialis, nysossatrina, kindekat, mhealy, MLQ3367, Matt118, Dr22, Aroswenn, yoink15, ShannonNN, catarecat, mattan365, Sh188y, el_guapo, Jeb, Hugh Mungus, Atani, adeline, JacksonBIlbrey, VectorB, immaculatekraken, biologystudent, SiddharthKrishnan, Wikos1000, Ryzal18, glamur8, brichey07, dmaar14, Bladeskiosity, alexko, Wiclara, epf13, Gonzostono, adunafresca, Sattos, JoshPhillps, castromirandacr, maziar-67, red1commander, Joeyiants42, Jesse Bourret-Gheysen, youni, jujul0006, Julia Le, mf.whitby, cgmoren, superib22, DancerTheo, AstroBwuk, bojanje, DarkSight, ngamer, mohnishgs, phoenix2050, natalia, rtwb, FA5878, DarkMKH, Skynet0, Devecseri, jenniferalvarado399, yoyo3654, Rorschach, 308drywall@gmail.com, adriola, IzzyIzzy27, mgish2, ritutrivedi, AlinaD, nacho90, jordanjernigan, h.kruchoski, gmiller73, MoMaDu, RanbowDerp, tho585, terrariafan, bwolicki, jimbob32, ayylmaoo, Mokaregister, Spudtagus, niseminoshiro, shawnguy, haseeb1399, DaveTheMilkman, Volonden200, YankeesO1, manateegrace, wizardmuffins, meaganhill, porldolphin, Charles Olson, Nightscloud2, tkc, Snowstorm100, jackb33, loulizbutt, somiew, bergen_paige, rebeccaloha, deathwalker, jdgibbons1, rajasg, claw107, ram-ok, Taylor, connard, Ikrobin, sir.lior, anishyboy, Reaven, eric278, Bastien.069, dannyj, MistahJ, Cherribo, rg519, varsha_menon_01, datboi2805, MadCowRNA, Ciren, nikidavani, ncpdanalyst, d382, CrematedCube, LOICVAL, joyousspider848, rgne, daniel the futurist, P1a22345, Than2000, scienceking, Wilglide, Anukriti Dey, Cajumasi, BarkingFox, puzzlefreak, Aikita, Schnapscience, paandragon, ekbergeron, BT, Attila Arslaner, WolfyTheFighter, fejrekab@yahoo.com, Peppersniffer, BFlash567, CezarRullz, biddikrik, Nino, hallb1016@gmail.com, Qriz, Parmenio, mpw68, bsw42, wrose13, Jason_Kim, 21.mariaelena.norton@carolinaday.org, kwee23, sleepdeficit, Aluxx, thetume, Rustin, renatomoor, adenk, poojakumar, GRune75, sierra26, sleepiechika, Calsapal, ANDYCHO, phicell, Joak59, cubrusso, sofialombardini, n8tron307, MxylynnMxrzo04, Queensalis, LonesomeBlue, Megaoptuimus, shuktija.sanath, shubham_patil, rohithp, BryanG, purnhagen, tanyatejani, wilgustavo, bulkhead2003, birds_and_birdies, Jaydrop27, Fluffymurderkillerstabber, myraelon, Kumatamo, eragon514, slozzu, rpavelka14, dinoserious, Dna557, adrewred1022, chocho, Zolthar, jpenczek, franz, Prysm, Harry Merrifield, Camhi, DJM_ch, thiagonchuba, Cubbydo101, jsicksate, vigilante, adrienne_richards, cbach2, yehielc, dagiles, fishchick7, keysmaster, jessicaangarita, WiseWords, sifu_g, Rodrixus, dklewi, accouto, Sylfare, Abby20122, lgroothoff, 50, walter1996, Sungolem, mzwee, EternallyGrateful, wacko star, jasongrey, Kingsley98, -Flexter_Dexter-, Duggan92, HarrisonWells, Quido, ltidwell14, Naughtoncolin, kirasiowle, jarrett.talbott, CheesyChaplin, angela.he.1, hoffmac3, 22itay, nickywojtania, reindeer, laduchme, CarlOl, pureid, SilentET, SassyCassie, elisewinterr, AJFILMS, yeshwanth, Narrexx, sissilessard, laughingjack13, MoonSlippers, 587003, ayana.shibata, HDCEOS, Kyosean, mashynv, gdeioannes, mallary, lebowski, ks, nmsarm, itzpapalotl, mdraper, skagel, robertalden, Jackal, duchesnesuarez, DaisyFat, slugiano, ScienceDJ, flom, tygerlin, Jordanacrlee, Brawtik, randomath, IAMTHEJUAN, lexivdh, aepollack, Toooooooooot, khendil, dyosifova, Luxian, rebeccadeangelis, deryny, Pixlow, sudachi, lenamolda5, JustaGamer, wohlleby, ttran19, Alpha10000, Lukedellthecreator, gualys, g00lywool, drkyuremphd, NotoreoN, margienide, Mizzrim, Delta_T, jackwall, jake_breaker, sahkto, DutchMama, biggiesmalls, Invisiblord, Scott40, Kotchar, zmox, deezedAf, MahmoudAgha, donotpress1, wadebrinton, karener, GSicknessTV, Patman2305, Pondguy, Monster Truck, Careinho, odile890, joeslick, Zanejg, abilamb, bigbiff, sylvainPicard, kkenmots02, PikCeLL, Meghanjohnson88077, crculpep, Darleenax, nightwolf7788, Xylon123, jcourain, swapan, sarahpo, dixsonco, Robotminc, hlmeyers, goe01, MIxtar, Bezu, clayshuffel21, musiclovers, j.hafer, fluffykitty1022, Kara.Johnson.1, Bavai, Maefic, Juppuh, marygonk, naomihogan, Dorvilus_B, samroyle, Lomqe, Genius1999, Sraemer2, davidgo24, tayaress, gracepaik14, pojoman, Syluan, mclandt129, Toniche, hamster, eternaviks, modayil, dicknips, cjordy98, tollim, sunjay1123, dielsalder, Reki, Ultrami, Kendall Horn, sprout, gyrados, Alexander1022, artholax, m.sayyam, kalexander, zhet, ashleychanpong, lucykatereeve@yahoo.com, edetpog, sasmtergurl, Cybernetic_Overlord, Hoopadrien, thejet, filwithjoy1, Jhuul, DragonairOmega, AkusherA, Supercoolman, samaracarterforever, AlabamaTF5000, pierreyb, futurist, remysanlaville, pmpham, LiverIsCooliGuess, jnikkel, hhild14, leeeezer, modziom, jezza1764, dddevindev, dosgig, VioletPines, gmasrian, Kim144, 1lyke1africa, TheLivelyGuy , Moleculejigsaw, cloudeeo, TheMannyMam, kittensrule58, mclem, auredstone, diamond_dust, Ezekiel Suh, mrityunjoy, ScotsHack, Eight, gilaadnir, AGioielliere, noemjam, Jaydon, achapet, JeffreyWu22, JeffDeaf, alexishill, kendall, rhyatt, redwood_11, sarahzar, elrbrown, peanutbutter, cheekyben11, piesandwings, derrickthewhite, TheBarkingGoat, batesh, dfour, lordemryys, SuperSine, IsaacKnight_69, ProjectWither, GamingWithLaura, 1venteiz, bobsledai, zad114, kal5791, mjortberg521, amitsahoo, KTQWestie, sueunc, TSW, gianathyaga, travis888, amyopara99, fogminster, Nynyo Sand, SolarNight, hana_hirano, Normal36, sosugicodr@thraml.com, InfinityRNA, darthkyeong, altostratus, harrytheninja, RAMETARNA, sokhovat, IANIS333, brewer.noah, samannihal, mael2014, turbofunkroosevelt, Y.PLAY, SigFig86, morgan.higbie, saintwalker, Nathstar1, Smartest Boy Alive, noblecarbon, hemophilia, iopi, exactly85, jowint, asher, pratik, iamthebiologist, nataszachef, tobias@giese-mann.de, Tmcm, Dusty1444, la petite coccinelle, howierd, Mrclownman, Narastor, kdembowski, spopovich, Starcatcher, BobTheBlob152, spiroszaf, h1n1h2o, taha2002, jeune faucon, kaleighspitz, 15minutes, ian.way4, Deuto, Jessicaransom, csgerlach, CDmc9811, fanhuanji, kotenok2000, retrat, Lady, laurenblaga, Annalie, dˆvsnubbe, Robotbrain01, sebols, emilysferle, bruno1618, AqueousPanda, Drewh19, dahyun.jeong1, michelleaye1, Jonsey21, Djurz, lay, DwarvenSteel, Trivia_Lover, mattghia, REMINITIONS, Jennabear98, Goats4Life_, Tha_Croat, D.Levos, metalmcfall, felics23, Gabby, petrosxp, deku_link, glace8, whenyouhaveadogbutyoureacatperson, cwong0319, Nikolay1, Edwin1, Noyboy, CaseyC20, rachelerinwilson, Ahmed Ramzy, XXDNabled, brudad, samtliga, guffy1984, TimBell, ENRIQUE, leaozinho23, Longmire, DLobanov, duchowneyg, toto 85, LAX4DAFINEST, HRutkowski, skoob, Doolap, mountinlodge, elicita89, adembo2000, 1scholzc, njia, aw, Hobbes1989, Itz Masterii, madmax24, brofessorSC, emakdaddy, mareDeath206, dchavez14, Happypeaceofpi, sfarris2, Squidoss, FunSize101, KrashHazard, Layla, sudipta0413, Bzerotin, MikelParsons9, albinohunter, marialominos, JJRodny, Jacob Hoy, pilF, JFLO, tombot33, jstanley_7, boky, GumiGumi, scb0044, aarntz, lord_haxon, Naldrun, Llath, jhhu, kristabarnard, crackheadbobby, dougdibou, Agrile, mister_fancy, Corlin Fardal, snickerdoodle, riedigost, kyrian, royalbtc4, evanchooly, Eos, Tatiana, clovercow, A6ronD, OsKy, ashokkrishnamurthi, RDLP, HD_thisguy, paulina126, Saniya Gayake, kinkajou, Deb.Tong, annettetsong, Sasirekha, lgfulton, Yasoo, peaches29, Ivan Tiniakov, Honeypool, ScientistMan96, SUPAH SANIK, Eli H, MeatShield, Asturvidor, naf2002, joey_h, tomasek, winterwit, semoir, Tigran44, vegas2003DG, maxfreeman, purringlion, r.miller23, Jokar1331, atomicsneeze, Ihmed, CelynBrum, zarzo2, rsealcoon, Anima, terraform, Pangolin_Ninja, gpj921, hawinst, silvermist, Bearat, Romancito, ilizzie94, pacoduran, DrainBramage, DanielHulbert, phillip.renton, narki00, pkp98, kmwon, tman555888, sma97, Aforgomon, jst04004, Jackson_winger, asshwin, camaxandra, iamnopyro, Xambonie, MaxedC, 6farer, monkeymolotovs, snidernatalie10, Inglip, DLoh, epunk_Andoni, sciencingsara, awesome_tau, Koerli, tiffany.kang.1, Rickyslayer9, IanAbsentia, MichaelS96, derousib, amavan, Zaelis, autumnstar, dawsonb77, nop, MagnusBrickson, Matthewfisher126, UTookMyUsername, Max2003, JettPanopio, thetrol, teahus, scd418, ameliemelo34, ro.pras77, BDNC, draster34, sunrisedewdrops, nadomako, stephane34400, franclau, bliblu, Tyromancer, joleen teo , Nicholas1024, Pumpingfe, konradm, cbargell13, benjamyn.jefcoat, tweedy, Roderigo, km94, Methyltheobromin, Major_asia, Etertna., gonza460, jorosco, hapkidista, MrMcCracken, morganafsm, micah546, ALOT of Caffeine, molly.minnick, _brie, kv53, karolina.pietrzak2, andrew.duvall, prettyempic, Sylens, aoveony41, ananymous, iSteeve, nata, imoonbeauty, dafrog, WeirdAtLast, reed.fry@ridgfieldsd.org, Sir X, baconportal22, Solomon19, JaSun, xna_north, fabulacraft, theiris, Shardy5421, sgrocker17, wydaniel, HAIL x 2 x Pitt, ca627, champgnesuprnva, DylanGames76, Dafitko, Cell(ebrity) Apprentice, ammydaroach, carolinerrojas, Aiezz, alyssaabram1, EPICCHEESE, Sweeteevee, CyberDemon, Fer G H, sullivankeegan, mbi, PrincessTaty, eoinmcq, Rin826, Kingjones, enavaljevac, Covenant12, BaptisteG, Sebelgique, camilaburne, Super Scientist, casmineg, Drrozz, HAPPY CRAPPY, viktron, hkim1223, Nthy, widder, Xenon345, Jayfeger, bravehp, Nachojim, Mesp, etern4, somechemstudent, ulfik, thefatman, luisramos, nightisday, pashtonw, Bookwriter, Hordvar, kylewalker, NYX15, maschakk, OptimusPrimerib, sokimen, Alana, xav178, LewisB, ROAR, Kramonth, Sung, cmccoll10`, kamoke, jakesosne, tagardner2, soccerkey, ToloveFLCL, steveycline, Irminsul, a.luma, meli.drd, superkuh, dank_memes21, Manroop.B, Elly-mao, may, pupseal, Wekzel, estradadrian, callmeag, Pysrilexot, lawmer, someonecool, 2016@eterna, Karyorrhexis, micha, cynthesizer, RNA4CancerCure, 303974, jjensen, Inevitablylogical, vcortez, gabriel15, CyborgPenguin, geekman9097, uma.kelavkar, aochengco, rhoffee1, Mysteryman03, Destmon, MacawSister, vanphilc, avia122, Deaddarcy, SleepingInsomniac, Jose94, joshuachou16, RNArer, ptlappe, mkebede5, kfeatherston1, kgodwin3, wteal3, Lsni3775, nast, Lou-Lou, nekomimi, jenfairchild95, epicoy, Aber45, ztokuno, RenA, tanveer, berna22, prociewbor, Cmscrew, PeterPan, LAB man, dfilias, delmo, wither122, i8ucl, renots1982, MOISTENEDNOODLES420, Cantigaster, spamboth, shollrb, seif, gabrielhawkpot, AleksandarM86, newyou, Aillinn, epinto, joepet, LudusPater, millb, DeadkidWalkng, veshka, Mr.Jokr, EverLark, ajpartelow, zois, Electra27, BaconKnight, hale6, ringo45, dianafedjakova, cacorderojr, AllanL, GenuineTexan, monipao, kedarchie, babitaneupane, mkap, Sanora_Ates, jman15393, Jade Keras, En_Dotter, RNAstudent, thelivelyguy7, ggcc, yasky789, scaff dem, eure1088, SpicyMangoes, Roderick H Yin, kasakito, alexno18, judy17, VintagePotato, 66jb, kateadams, LilliaB, skytheasianer, giis, Isabella Rose, TheBeege, YanWoo, gkramer2, CaptainPankek, jennif3r693r, pojko, karenlin123, Lgrace3200, gabirelis, Nashova, miuosh, harry.w1, mporter14, dnguyen14, CatmanFighter, nuclous, asterisqi, diogocurralo, kennyiscool, Amargi, Aj579, kitten, anderson1116, slovenka2000, crooksandtheives, IziFox, Kyurem81, rogrido, Ase10, Airlegoland, david20090523, LRaske503, gaetan, 186879, Joah_135, Vahme, sks, yassine, kennalynn04, Doremiflustist, eternaGamer, bwabwa222, Fallontine, jv0535, violentine, agentpixi, the_sum, Jettaleigh, magikalex, NinjaScorpion1, PedroRibeiro, justanotherboy, hydroxyl, NiCramer, yagod, GCTB, Magna, Sushi Cat, janeisakaye, Sapin104, asilentwolf, rconniving, Tseng Alvin, Vad99, hashim, Qpid, mace, jww243, riveren, fearsomerooster, nbwtf3, tejaspchat, Theonintendo, nicknemer, achapoval, lpusic, lahein1970, Paulgreenway, pmbrooks1, Raikore, ojmao, addt, lewistodd1881, isaac.z, qqq, morganhundley, citizuser, selosari, willswish22, TheAlex31, htaherkhani, emm.grace, nennie, EGSD, grays107, Nick Sousa, ShadowCube, StepchildoftheSun, joseph_seamons, Dantara, Wolfram74, qCricket, hcurrey14, crazyadz, nicolasfdg, Ernestodlc, N.poox, Blinkerz, colinjmcn, reidrodenko, neefer, Elfy, will.colbert, nickbudz, wyshugart, theresa16, mkeller7354, smarthuman, rmaloy, hashemsalem, jackdog45, svanderheym, Jaixmemly, Renton_Innes, apsk7, MorganClark, patrykwielowski, callywally, arangosergio, sequoyac, G.W., snidersniper166, Agk2014, Phoenix26, jed122083, Semored, maxispazio_1023, AJT333, baileyhirota, elias.erwan, Takos, Emelia_xo, nancykz, Ayoin19, DelToboso, erythro, carlguack, fjs507, elsherbini, Astuch, Zageron, YelyahNaloj, amberdanielle, feli0922, j.foster, popsicle2019, atzi8360, rpmolitor, guido99, mcdermer, shooresh, HoodTinkle, ansonberns, 23chrjam, ankitrana85, texankaren, achilles1205, floyd, octopus1414, moip123, Tatane142, novemj, memesanddreams, jvoli, annemcnairn, test535, Xodom, kirankmr, myceliums, AgustinBrusco, dnwilson1, kcarmen, packa, karim__2004, BobtheeBlob07, DoctorWho16, Xavier Wilson Lit , momoclark, RNAtestaccountGGG, cardsox425, Kcrawford, marisvalentini, cashcon57, sigmundrs, sencha71, Ftkalec, richpopik@gmail.com, Tonyshia, Seha, TheItalian, shifterks, grillo, MASTERDERP, Donbo, AurorNayt, DragonL0rd132, ima_cure_cancer, jlee7, IronEve, jacob.libbey, Febe, Mathfrost, Claudia Vivoni, kristynakulbova, bdaniel1218, waterbear, sthornton3, Lari, TaTsE, saffytaffy, OliverQY, shudson10, test515, justksketchie, Jomuel, klapm, Jackson24681067, Fox_Arthur, skodborg, TD23ASUS, tudunk, Goon124, laniejung, idelmuro, mannylovesmusic, patrickseed, Nic622, mouseofmadness, potterland, jacobk24, j97henry, alina.fabyanchuk, johnskim009, 78904565, Elarim, elcaptain, Silesor, Gp3115, tayloary000, Astrael, maceyway, MarcusIsLaughing, jordanbonilla, Azor, gravi0la, DaBurgah, marteden, adkuiper, ricarn12, mortrek, matburton, aadevries, Kappa_!, El_Gordo, Rcook14, ntegrtytildeth, MicaelaHurd, linneacookie, Sevan, greendoor, 1grafc, cdsproles, WFLO, malachai, Percievel, Fishy Human, grogan, atla, jennifer.popescu, maxic, warbear, darkmoonrising, Dr.Tentacule, adilliot, joibouphaphanh, braxton779, kcin2001, dracke1769, Litixsam, Hydrollium, Bobutter, richardvahrman, davidmj101, SkyWolfy, aturc, loverde89, trutruc, Vonkensington , g4bb, photonjack, biosos, gavingavinchan, pemabro, EmmaCy, jennythefennecfox98, darkcoolboo, kenneli, anbailey, mindofmystery, elnirath, ishhhh, marcohern, Varoth, Jenna2017, jorge23, weskwong2, Allymoose, LilBluestem, serhat, BobGamer, vimes1984, dacp, jelliott14, Jailine, PMEANS, iqir, Lejibet, schaunard, CMOsimon, oraniki, starbort, iminto, WynterMoon, rpetter, benckx, casimirth, JohnMcClay, sgarrick, bouriquet, cabezablanca, ratster99, pielikey, Connor R., bubba ganush, Neuron13, rng159, jhyoo, Codexys, dianehardy, hotcreek, Alexis19962, Mr Fusarium, Melanchthon, BHooper2016, ek7opa, Bsnliu, oml13, Nicolas.wang.1, emoglia, [Haku], cobel, bibi, Aixtzr, catpoos, Tooth, h2ik, mrnat, roxanneheart, robolobo, raptorrex666, AllyV, JAILINEEST, aschlueter, rapefu, Triste Realidad, 118af, TomDiderot, klodrik1, fusiongrl1115, HCBIO404LHoffman, MagnetoK234/rna, narayangill, Lumpa9871, chris2b18, D0rsug, acloyd14, cgandon, chemnerd, jkehoe1, aresetar, alsup, etseco, kforce214, julywaters, alecrdj26, rachelt27, Park, Mehdi4719, CC314, Zaneta, magelan913, ilerm, justacea1, Kittyhen, znep, Kuk˙, 1RNA1, cosmoletchik, NBG, BobTheGreat, JoeMancini, Neal the savage , Rayzapper, Fennex, jlhanes, Mattymatts1, ZRussell1, KthulhuFtaghn, DrStrange, openhairpinleft, 1245678910101010, test1306, fritangas, Lillies240, Bigginheimer, gregrhunter, BENTEN, GerardoJara, mfrenz, anitarobinson44, bobina44, byolog, amparo, mclem1, Shivani K, nogoalz8, warlamb, MasudaSharifi, alexxiaoxiangyu2005, cussoandre, Kiraraud, Hassizzlee, lolamarie, jaweghor, KarishmaReddy, SleepyRat, kirederf19, thegrottman, THEKNOWNMODDER, daWinshu, marknever, Felicio, cocameli, Evgeniia, MANelson, JBthesciguy, esmurria.a, ayowhatsgood, bOip, Ms.Mustard, Vinceble, simran.garg312@gmail.com, RedInk, Lilie, 1gr8penguin, rdew, emilynguyen, isaac.sides, wdseth, Awesomefrost, Romank17, faisal254, lokivari, manishsamson, Buddy5534, mitchell232, pavel.wohrizek@gmail.com, 17victoceb, at0m, Margotofeles, bluhma, Davidcwalls, hizon8, dhartman, VicHimself, joeywoneill, dufdufduf123, bgyinr, Rhzoid, lepoulet, Salveur, lni123, alexannan, NestLife, sci_girl, Albert Darwin, LouisBarrett, SuperEscalier, Jordanjohnson, Haantzy, Le crÈateur, slack, agbunag, megazarbiman91, LeORtH, dmrmay, Sinichi, clarkrm1, shester98, sana.quraishi, Romantic, alimaverde, weateaboyatsea, SC Cyrus, Preston.smith75, AndrewYu, kathys, SanthiyaM, brittanyrose920, Novamonk, ahzdii, GeekyOtaku, dallendanger, west222222, buboh, clr, Zegnar, Adua, DRPHYSX, madgehugo, Dado, anna.mikolajczyk, BearonvonDeathskull, reado, SaharahSarah, EYEYEYEYEYEY, Joas, steven85, aie, JJAndrews, marius2417, DzikiZiemniak, LigthningStorm, precociousPathos, eren41, dardalte, zako, loop.golez, jry2001, Gerardo12, Los Macheteros, Kahless, Adiictoo, WhIteVIper83478, amikelov, Bubbles667, tonnio, 2017rewphilip, meganhess3, tiou622, chromebook11, J*, Nicholas Mephis, mar109, olivianeyer450, navw26, The Lettered Olive, Chroev, dasgenre, smithj, Endymion, Jhue, reneochoa99, EZAardvark, AugustS, pvincentruz, Professor_Cortex, Veronica Soler, Openfire, RichardMorris, jero1717, oskarvonephesos, hentrekin, Dipakarki, jennielynshapiro, polishben10, Tenebris, chloebeard, yorky, parisamahmud, jacobkaiser@gmaill.com, Guilthunder, aashna.g18, passeurdemonde, sudobutt, Connor0323, jcseward3, MySecondNameIsMolecule, Drashti, 01014750, xXLaPetiteMignonetteXx, samamirfar, qteapie7, jaden2000, GrandMist, jdigeron1, e101, akz191, crystasugamura, angel.yi.1, ameena, Jbuff, Kyushi, lillianatlee, dragonjake, Zelyn Lee, JonathanSchroeder, bwil, Bryco, sterne, SigmaBetaApollo, huthut, MajoraFedora, ana135, luc1s, manitu2000, Carmorik, LesterTheDolf, TheCatsMeow, czikajarl, MissRead, KeiranN95, mouchix, Snowywaters00, Adrianna, lewie, norvegrland, Dawngirl9, Hawk680, nvrogers, Metroplex, pirete5, Meghaford, drjobert, KrzysztofNowak, KileyB120, demonicsteel, Paytonhilling, JJandDjango, Vidar, shademan11, joss33, BearCat, phillip_phil, kenda106, madrover, primus09243, Aminoquiz, nebula_point, ragnaric, abustos85, revarcline, Isakswe, MolybdenicCrystalform, msommer, Dugg, cjack22, mmarcus14, gcwingate, wong338, The_Geneius, lilja, SeattleCRW, abievanss, Jorelly, YusufNM, morgy, Hoanguyen4202, goolywool, episcopunk, setsail, Bnanishu, Mav.16, amber7malik, anarok, DocBrains, tiawhitlow, Edwadosan, DressageGunner, BoredBro, monster, katarzynawasiak, jacobedwardkaiser@gmail.com, makises, supermarioglitchy56, SamboBro, gmalfatti, MoltenRock, trookey5, Reece Jones, Merifri, gaja, j.cole, alligrace1234, Maustschool, bbc001, budga292, DiegoM, The0therBEN, marcosbraganca, jkalwa2, joshuajohnhill@gmail.com, GoldenM, tombarbsand, gatherer11, superloutredelespace, eyhjiulei, The Z, Thezi, interalios, klc8272, tissana, GabbyHoffler, mleighton, hellohumans, Delvis343, CharlieW, ThEnlghtnd1, MsJensen, shacamin, Josiah94, cwdavis122, ghujka, frede115, shoaib131, dmd, Darth Kyeong, Avecchioni1, the eternal creator, Teykana, Alexander.Tashkeev, antony.corwin, Secboy, netik, juju50, RIdgeC, TheDarkMark, lorettaf, filnias, awatson5, Mahmoud, xohorses, royrschach, fatima2016, AliAlireza, sinanbaltali, zoemote, feffab, mcginn1234, EIMR, nancy.shaw.1, anoano22, TigerRaichu, Dome, richardmalo, uokwuolisa, desarianhase@gmail.com, DiamondJub, Langolyer, Hortbek, mizuchi, freshkide, TheRealDeal123454321, mincerafte, Satruqui, RondezFox, Dienonychus, test221, Cyba_Forge, AnotherKen, willjones1989, bribri13, Buzz1215, Bharadwaj.M, titidead, Adry_1, kkalwa, alph, MFG_hankie, sonono, milkymanda, mendo200, papageunu, DinoNerd03, bored2death, andy12, han.zeien8, fbraccini, softball_2315, Fyrax6, Nexus227, wrhc, jhuber2, pavan248, Ian9, Straw Hat Luffy, toich, jadrien1226, TheSuperDash, HellShade, kkelly4, rashiel6, emiliano87, doorocket, tspencer3, Cameran, Buster96, XtremeGming, corvus_obsidianus, test2903, stock21259, JumpinJac, ocus, WaterMelonRules, dorinda42, jillianxjohnson, HelloTheWorld, alnoperr, gloot, JColdren, Laurasgwd, reapercat, Kobrus, Littlemisswolfgang, Chxck, janeyd, lsnliu, Mrees8, vichawkins1231, brausch, No1ZeldaFanMan, TWLCFoster, womble dung, grandest, Itom, zombieoverlord, 5166954165, TardigradeNerd, maxharris22, eikcaj, Odyseus2, Xinlan, jmcmullen, Patola, kendradavid, J Woodburn, dasoro, ianniccheri@gmail.com, pirate123, Pkitty3, nwiegand, Rico*****, Pinkman, ryker, jahuck, HayLeeDavis, ArnoldS74, Zaelix, leipzigRNA, chrlrys, Tupulointi, Justin338, hiouf, Tyro, afhollmann, Kurson, kainoa14, phanu, Exitron Apte, ugolee, Manix03, cuil, Googlichou, npshapiro, marioistheworstteacherever, Sultan, Mememaster, mallow, entropicmonkey, julian0223, whatnonsense, Braidweick, esued86, bo, shaebutton, Spreden, Xenopsylla, IsDoctor, Povan, Nougat, cpfatigue, judekershaw, faliah, wren6991, Codeman5199, shortcutter, nuwar, lukasthesmart, Hxcking, Frelghra, Purpureo, BLTaber, TheMagzuz, makonomy, LiddyNiki, vytran1629, onyxninja, JoshuaBioinfo, 7896542a, Tomato, AmelieMelo, ellenbrady, Dr_fovea, nmohan, jenkinszdj, diegosuarez, barney.tearspell, osch16, SunSimiao, hare, Innerlines, GreatWhiteBuffalo, Anaxagore, koziel, Jbaixful, ragnabo, CarlosF, deprezke, evharte, radecule, DanielFord, willhong101, mrmola, Stryke, makayla.haggerty, emckeown1, pejowei, Daniel_fasey, nailana, minerva66, Ana_Gabriela M, dboy1015, Haojerr123, juan cena, jeppettoh, KAmrine99, irenepellegrinoct, PreKrebs, norseboar, indigopari, justinhuu, Sanchitou, starrmyrtle, deep-venus, uberkiwi, RNAtwister, stuppid420, Nephelai, ejcarino, Laureatka, Bio-Robot, hijoscontreras, signlover05, Ilphrin, Materistic, monaxue, Ana-Marin, cptredbeard, fjade, kedwards13, kbrendel, Kurkio, pagec, grace.lazaro, GMillie, conorjfhowe, VictoriaCappon, ghery, ehs4n, gardenpea, mgeistler, jigishjpatel, syfygirl, Acrylude, JordiSM, fsbx, IamaGOd, shinymetallic, lastbluerose, Gladway, pluton31, brannonlynn, albertox, jaykapadia72, Infiniti1, mer135, RedSpah, Beat Em Bucs, Ern, GetzMonay, DeathPulse, yukimitsue, j4ckn3sia, chris_topher38, mswhipple, cjsprogis, CraftedGaming, rick1689, csherff, rlin, MAZEZ, muscovymischiefchick, KB171, ecline, happyFaceScientist, Alec.Santos, BlueFawkes, coolmariahamster, mgp0127, Aston101, ShadowMimzy, snaranga, restigou22, Dazarroc, gribskov, aelahi24434, jedikidd08, AdarDasha, Poitier Stringer, Atomos, kperss, justinberryCPEBio, fedeg, kromaine1, burak25, maitreotis, AndreyMozz, ElvenNecromancer, kylekyndal, Artifexian, dureshehwar, mediacodex, dlenart, TitanStorm, totor, Classyklutz, cstb1, Aspeek, EdibleMetal, logonxix, cdpeyton, Bioskeptic, boxchanp, Magikakarp, pieoflords, mikecody2, Kwaters1996, dclone2, Dash, jaguar98, acs1972, alfa2, werehog47, sancm14, Wizzdk1, mcie, Maevell, AustinJamesTheThird, POSIDEN2, VastianZZZ, Dirctus, kimnos, xxDontPanicxx, LenaMoldavan, jamesdemchenko, sadieoflaherty, voldemorder, spencersimko, Urbain , OwlMage, aert, davidjm, tutu2666, Andromeda31, sevoro, playboy78, dpfan42, hhentschel, poober123, WATRDAGR, DVSJonathan, dawezzz, SwimTeigetje, darkh, anthony1, jyaworski, nicole.josie, nmeyers, MadCowDisease213(Jeffrey), sergiiomadd, timbio, strayjtu, Cas13, jokafor1, Jackie123321, basg, defectiveDemiurge, cindyzhang99, tanabornstein, Furlucis, deacey, atrain99, NaiF143, Chenrezik, cmitch819, testtest512, Catyboo101, Ilrae, mayrobe, NoraNeko, andrewking203, KriKriBioChem, adecks, skulls_4ever, leloucho, tatalex, test8114, JackTheSlipper, Rdilag, degracemm, rbierman, EpicGentleman, BenBoyle, jc31, b_howell126, cienc, ngrous28, LittleSully, Hesham Webas, mrtrules32, smashy, mhemphill618, javort, nonm, testbarney1200, flipsyde1, Metalbeast310, Sanjana16, jmelamed, Brycebybee, Jaysonium, pathrna, davidemowry, NLDeMino, leoweigand432, ham_shoes, acon, liloute mauricette, hjbrouwer, IronDoc, matou26, Huldor, dipsy0720, sksbell, FatJustFat, boby, lecalaza1, lcs577, sinan, Dennis Ward, Medic2017, napen123, amira adel, Shiroan, BrittanyMK1, Smirfyman2002, gatortor, Arenwyn, queenlilith, terblanche.jaco@yahoo.com, CurlyGrapes, jfbrennen, quarkmarino, Bean_Fish, anthonyseaman, Krischan, alandiaco, billydaniels, vilmanke, adrian.pavel, REAL_POIRIER, Obaid_123, gauriketkar, BarbaraJohns, samanthapete9, Hyperium12, HayleyAxelrod, katelinn, teyhla, LEGEND, efuji, Lucianaville, damien.choron, macas105, aelgammal, gundus, rdthp, iameternal1, mistermurfy, BrittGalante127, Ariana1012, Guy Shaggy, derivan, Asj96, amswid20, emmaokapi2001, NTH0005, undeadcowboy, firesparrow, yellow_blacket, DarkMaster, Windayme, grace_face, krissmennell, lizzyleonas, TsukikoCurrier, azhang21, b13, SAbeannie, deadtired61, shail, lptaylor29, desired_username_here, Videogamer70, jcreevan13, test423, davidn73, udeudeude, janghyeonggyu, Blinzzo, Akmmaher , simrun.ursani, dong951103, sadpony, MRADAMS, SkiaFox, oiseauperchÈ, ShyMagpie, mccle132, virtuosummer, bloxbox, jhabtema, rhartogs_eterna, lwongsensei, migythebomb, R0obiin, kat_w, choffman69, nana, zoeyyoungg, sarge, HaotianQ, Zero Progress, Gainsboroow, PaulBrett, manyu, DrGrunty, mrocland, agent3554, tunrau, Devinm311, boopster18, romeubpaula, Protean-above-all, anchuu2243, Xavienth, asebiani, HammerChild, teodor78, mdietler, arcanepikachu, Karmito, oweny, msampson7,verizon.net, Haartemis, Robert2017, SupermarioMLG, sabersm, Thanatoons, Bilal, pramod_recode, vbenavides, Pa1gemiller, BryanGmmmmm, TheBleep, arshad, ingressbetauser, sovinirs, srdavos, lamsi, etsulliv, Profa CZE, slutzy69, weinsm, 1stevenp, andhrimnir, Nuno Marques, swagadactyl, EHSMiller, TopHattedCat, Amira, DavidNatanael, ferg, Lotesse, firesword, zamfire, loukouma, kdease, PoorAnimal, Eirah, kajellereth, watertank, Belga1, hanmaldo, Zonner2001, Samoyed525, Fantaisie, dickeyjohn12, ikraen, a.ahmad, gogata258, fermion, sunnyswanson, abowen24, Sandor Finn, amneto, AKSCIENCE, Whitefox777, antartic, Ch4osknight, pandora, edwrdteach, Hector, xylophonw, jemarroquin, redcat1, _Maximum_, Gzarl, mr_imaginator, wallerc15, NitinG, minehorse, dreampie, silverknightzz, leegaarda, micia2111, Doran.Woltersdorf, Geredd, avanderley, NanoAluminium14, copeland, TroyIsMetal, Red Gab, marktrofimiuk, SB52, Table5004, TeenageTimeships, Jojobarbar, CarBoysFan, samoapolo, Yoine, PedroCunha, bk1010, villavo1020, eviljohnnybravo, 186095, tubarocks, yf.o, Nikita Brukhanov, young duke cho, SamuelTSO, Zink, Izenibad, Wreck5tep, Ian Day, DL, fulikalter, azlen, anynooone, MineLinkFR, mariam25, michaelmior, izepeda, Hydrogen2Oxide, lucas.p, VanOutsider, mayorale_, nkirkpatrick, henry24, Sovian, poplarbul, Dreamer5959, kharkins16, Atherne, lordotw, marcus42, nathanK, Fire875, averde, dragonwin, dcandl1, smccombs, erudium, Skillere, kaelync, mezzol, lluks, mattkobi789, emau, dgrem11, Fg95, Pengwyn, Hippi Nasehorn, bill nye, drwibble, Antiquus, Gagcha, dfriedland, jfowler13, nlstudies2205, Magic, kurujail, TheGoose, EhTee, Gabrielle, Jathany, kuhlkilla, Flodarius, amarkus, butterz, ByggareBob, TwistedTheCrux, rainacorn11, supodin, Hyperion203, WafflePotato, hammolan000, SRain, mutator101, nmcnulty107, K-Train, Sliderlo, jackie.kephart19941, mahdi-g, Rchitty, beto9202, maiya29, nichti02, GameFyre, laurenm544, elportugesh, mabs, aiden3dd, lios87, mpena1, JuliaMit, mwilliamsfu, sarahansullivan, shu, breanne.ault, cydittmann, chia_cpc, Onesmy, Kulpas, drkenji, tomoedachii, krondog, diergotarman, fishfingure, skandel, geizio, Ayex, Ares_4_Life, mariomaster57, djladancer57, vhrico, nailah_din, AnimalLover022406, Dr.Pizza, HPApples, tcfreema, Pe-T-eR, taehmmartin, shay5222, Isilme, Savino Falco, oioipo87, khatniss, atmorga, Frankp2491, LordFlashmeow, kkohli, meka renault, loxie28, gilaki, Obliothing, Zagreos, nonma, mdf17, alx-001, coily, jimbojimbojimbojimbo, goldentwo1234, Jukagiso, odeiiis, spacegandhi, comatics, profbiot, 445295886@qq.com, eternal sreator, exposer28, Noraip, Tsa6, Nihilanth, thatguy010, VenusRain, Snake2417, ShadeTreeScientist, Jideox, DoctoX, AadiGaming, gjg3210, colinvignoul, ahua77, Nickatnitel, emmailinca, ellis183, Cal_Capone, Playaz, Scorpion508, olha_vypovska, ACRichmond, srmatiol, GhostGuy, kachupyn, seablue1234, digidude, marymmm, beubeux 74, Platinum_Hunt, Happycowsmoo, valto, addylaurel, chubbz808, Alex132, skippysk8s, allynharris, jingle, SkyFinder, s97, einek, Macbest, dubois, snelleman, Its_Me_Dude, sstrickland69, efilippa, discombobulate, 20170203, xerrix, UnderTrashLady, Catlinv, Chameleon12, dudehdrjs, WagsTon30, ChippyofAmerica, arielm, Xencaye, I Like Pie, MattKeehl, ByronH, blackice, queenjane, mywither, scottabutler, Tildi1, zoniryan, Miguelftw, alohmann, TRees1999, julesverne, NikosBlu, waly206, phizuol, Khemistry Kat, Antony246, chin.nems, Muzozavr, jasminetofu, morg, allaboutthatbasepair, CRIDRAGONS, hsmith16, katakolm, Becks, MormonJesus, ralphandeli, VanCleed, 41cy0ne, niky1, paulserch05, rgeheb, tolland, ntbm, 422201, Maryam Ahmed, elemenopeeo, soadreptiles1, ShunTheElk, NeuQ, LuisDNA, smflanch, nodir, .., skky999, Julio974, hebjo, shakahapa, nstephe2, sonikiliky01, kleptine, JAWS1275, duygu, moli, Stecj, Faitmaker, SuesakuBlood, jrosy43, Josef D. McClammey, adnsalvator2090, annika.chan, LandonZ, trueanomaly, Pario6, daviddesalvo64, Nogard Noir, Senga, dan18662, Heinri, hypermechanic, amritha.r18, goldentreefrog18, hanxyolo, Briag, Jacob A, SirDusty, kcarter2016, AccGaming17, lee737612, TROLLERDOGE786, jeffledwin, cpezz, Sarahi21215, nikesilvermoon, earnold1, lunarbug, SultanFX, photon666, OrsonAround, zh69, mani, josemar, hyphenbash, jepe, rapidash, Emma_Lindbergh, ganipa, rey3002, oly08kckr, Gustavo, ivypaige, bananas are yellow, Octodon, narezul, jocelynrobles, martijnmunnik, AdraniBelegil, roaneskin, althusser, SquirtlePWN, pOne, usiadia, Memi, CRIAVENGERS, ailenvittor, kennedyball, Christian Tsivouras, adamatallah, MuteForce, Fra Frusciante, tsteiner, crazzyboy1219, ivan390, MageofMind84, kiksmahn, TheZMan2960, Espequair, Douglas, Sunshine0, rmon, JNJones1, M@lice, alexisrafael, yuvalturni, ptismall, pazelle, faithroz, miller6790, TheKnack, carefreepenguin, kthakur, Dr.Shadow, mariellanewell, skyeboy191, chsevy, gamador, maek666, UsurPedro, TobyOrNotToby, monkeyinemigration, parski83, ohassan, sherff, dardenofeden, pinkpanda05, daniellejoelle, sfmeade, Crissy, jericotyler, mnrkrs1996, lamaral, Caleb99.0, bobtehgreat, RainbowDx, test1080, TaoTurtle, laurentc, ginomio, quantum154, bern26, wbt, DATURTLES, ZenDog150, Damiano, zhanghua, TBTD10, BiseDeMetal, aoibhinquinn, phillipsar, Mayacny, Alma, NewMoose, derpitron 1000, Hairesthai, abbycroxs, UnDeadcoyote, jisaacson14, fyc2108, genesis11, paddy_o_furnitr, coolkid69, CyanNightmare, CXRom, lolitafermion, nilloc171, asorren1, Kojak5280, turloughcowman, LegendInShadow, Eigam, Demonio_mx, Eikyu, Portgas.D.Ace., tredegarshea, MysticSilver400, tokintalisman, Antiepic, rick shaftington, daxmell wutt, dakota_c223, hunter.goff, letsdothisthing, katwas93, test1380, Antoninon, paola_jose, facial, reginashkreta, Rafe, AFritz, eddiefranklin, ashrivenai, SGSalem, Huraqan, anura.shrestha1, K_Berg, drkill, ghoston, EverLark13, crilab, EOD, gf-test1, Po, rna_farmer, Quanican_Bleu, paradocks, neverlavender, isaacm, aHumanOfPoirtland, berserg1, usarthur, Jlewis, arcamax, sturgeona, supespacerotter, seren di phytie, Echoech, Kiracutestuff, AndyBloop, ride2946, Dpirie76, the scientist, kittens99, crisis111, ycarmi15, MedicalScientist, yiays, nukers473, Killabeez, GretchenG, jasper1234, iloveme, ghost23239, ris23, mccarthyelizabeth05, MATHBOY12, rapunzel152, frocto, OleDegrum, resmander, Mouradif, nich, Golbezmeteo, Telvarin, marcusblock, Tsolstice, Bragolatch, anica, jrthom1, flopau, itzpanda, SMDCharizard, Luis2016, Icarus_123, etinaude, sun158, test12525, brandt.weary, Aku, lxlarkin, RedMixTheWolf, ricizubi, Sanctification, yokoko, Arlnoff, eladiodr, Usmed, waleed_res_ainshams@yahoo.com, otebios, dch10, moiz16khan, BentCookie, Elijahcarter110, Skalagon, Sisoma, Zhenya232, drstout, makin, kouzmenkov, Dax Mutt, gacarson, EO97, RnaLilly, behanelizabeth22, Sousou, WaWaLuLu, Mechpanther, marckristians, tubez_c, OasisMaximus, jakkara, amyleerobinson, 7ships, sathy13, acurtin, shanedsouza1, TheLonelyEcho, randomguy1234, silvan201, bluffburrito436, bgohan, krshridhar2005@yahoo.com, mjk2221, sanya.rehman.1, miniverse, AvaCassidy, dantoto, AthenaFoxx, spinodalk, AhmedAyman, 0smallwm, MrKagouris, MuFuChu, esyang, kroquet, AshtaraSIlunar, Wounded, Etvader, Gerb, Skriff, DRDNA, Eagle7996, AntNGira, Sans, BlueBlaze, Tolka, emilejeanmart, melias9, demonsorrows, LateefWalker12, armanb21, janandraczko, mattdo66, HGMACKAY, pokemoneterna, Milk1234, ACatNamedFelix, roaringlion2002, Lindsayvolley98, CgW2go, romelsan, steliotesd, fkristina, marwan, timboboss, MoonPaw, Gustavo.M1, Torghal, Ainsley, SolarDiamond21, mehmetesk96, Kaldrick, zsuzsanna.gere, Saml64, monkitten, jorgeisabadsoccerplayer, eclift555, willdavis, XenoZCooler, dpedds, Dommos, dfpe, schwagerjjr, vekysya77, tom d khat, moralm2, kpedro, sonicmiller117, Wojtek_Domin, muse51bt, jhnkruger7, Uziel33, rexny, jcmusterman, bobbyb264, jshallcross, Kindama, Pocky4141, fejao, TSkorupa, genetix, Hampa, johannesjuenemann, jonturner, cemangini, ElephantHeart, alyssa.anderson, tanguyofgood, lamcho00, brisingr2001, Sof2929, hgkamath, Baatr, wrch, Sig, andriykopanytsia, larry25427, 13turtles, nick1243, annie33, EpithanyRae, unknownME, diegulu, FritzsHero, myrrdin, DavidHan, saraurier, Jimmy P, matogoma.meite, peebs, pinkunicorns06, devonwg, rajkaur, gabriellecrivello, mopman23, fetiola, alextheboyzander, IBricchi, f-stein, socky1234111, kubica94, necet, Sthefano, mcass520, ash greninja, ingwa, ewang125, davidmitchell12, tomduffy30, Jayman, Penga, 598905032, DomesticusRex, Torrence999, Aceofkings9, ethangmt, GrimnirFaltz, Jellifizh, thepianist59, Whaty, AshKetchum, arexw1, tarik.azouz, ruetherford, captain, ErwinDurzo, bobbey, princesscarly28, KareemDoesMc, zoz2124, vicioushalfling, delkaim, fatimak, dnewton, prismatiQ, yekbunbihar, SubaruSteve, WilliamPesto, hudzi, TrAsHeR, allenwalker, kptkurczak, atrus7, Iz888, Assaulted_Gnome, dudelpudel, torikh, spencer1keane, dmtdmt, bancoesa, himan.tabani, DrewA, eol212, Zence, Lucane, THOREAU, Doller1, scarr9, BlackScienceMan, Homebrew, jonathanwade, MonsterMunch, Needlepoint, tspencer, mkaufman1219, OG-LOW, codeblack77, nicolebessette19, Brussell11, mercious, AnnieQ, test6356356, ma529, blu_blac, alex1454, samieru, OhAEg11, ulrich0810, Emericx, angelD, FlargleFlangle, mwave, ainsley.ballard, BenjaminN, Paarthurnax, axxiomm, Leoco0007, Feline Dragon, Ricardomd7, Chris63479, joelc11111, Maestro31, clemclem06, Tucker, BigDaddy69, Karategirl80, hsgdfgahfafhahfadhf, Vladik, reefishes, robertlyan, dhruviscool, Lifchitz, jagodzinskajustyna, ProudMuslim, AmandaMoudy, wwright, demenkodavid, matt429, jgraff, jwilli3510, kuroi13, Timboskinz, sharksting, aaronconnors, rednaskela81, Jeff35, Jyotirmay patel, signguytodd, stef of earth, johnsonc, Nick Fleury, stevey, vutrangyan, Davebeevee , jujuzeking, Skillzdavid, tday93, ailene025, CreazyMintaur, ??????? ????????, lukiv, WebsterWolf, ccormack, HitSongs, saatvikkher, cxndxcx, ReillyDeffendall, joel951, Doc Roberts, Kwokodile, takagisun, vanessaballeza, satishbty, lydianb, MuseinMotion, ValeriaRod, antonkulaga, gaijin1997, jluther32, Fabian, summerluv418, ddesalvo18, amomarie, mikka_is_tall, Kenyana, RainyBei, coreyday89, Knugen, nathanh, the fatman, rlgcitizensci, amaccormick, sulfolobus, leyl, octaviandavid, cjacquemart20, garrison, ScarShadow, rebecca.dake, giorgio, spymatt88, allasophy, harkiran, Jennifer Pearl, hm986819, Spartakaktus, violetnebula420, lanny20033, j.science, Moody981, jasperjohnh, Marinelfox0521, emelyyacunaa, Hashem, coz, haugrhol000, MartinSamantha , whiskymisky, silkydoug, PsinkaJones, a.mcelroy, Ivansx1, mockingbird, isaac.howell, ps24jeff, maxmax, Stinkles12, mjsbgold, kzazar, hamid, kevin.heinecke, Arcamorge, Grabrabriel, avoliogab, padidehdanaee, Lukia_raregrove, oldbigal, leehotz, ricknm247, AllieGirl, sambea, kyidyl, Eryn, ode58, memoaguilarjr, yoffset, SamCat_14, Zacsar, andersonvom, tiGer72, bacon117, JuanCena63004, Schulio, chris62, KrystalFrances, 18dana.fowler@myprowler.org, jarednat, TrueMK, MacGomeyBear, thumperfuzzy, suikun245, Hagrahell, cesarpagan, IconMaster, AngelBear, emily_sch1, xiantk, ratgr, Dieguito, matanana, otmurray, Tortoise, anibot, Andrezitoh, bluecombats, thekijin, SmoCoco, aweidldl17, mabragor, zohan1922, Neel2000, free2choose80, PalChumFriend, sydneyelizxbeth, RecklessCaution, haynes056@yahoo.com, Pablodb, FraggleZilla, r0bert2, DavisKenney, PotatoPalooza, joecap16, brunamoraisv, dilcannon, nate11, ngiaffoglione, Perrine567, molitor, cock-juggling thunder cunt, CoopGalv, leshroom, JakeGeffon, mnemogui, Nehpyt, TP3413, rokccnfv, matteovinci, psychologist, Shadowace112, reginaenox, jeepwran, WitWolfin, fatjedi19, alexandrujuncu, Svenyon, kdc898, Fantaisia, seb1, eflan07, cianisasmartguy, troyang3, Vadkoski, test22222, Carita, BSOM180, LFP6_Test, dhbgirls, hahn7301, bubbalex, tnurlanov, srspore, bascule, VanessaCruise, mahmoud504, fauxnetiks, Griffin01, Revydestros, kprotoss, luismuniz2016, patricia, upinsmokemon, neilc, Lilac, dpfels, npnelson, Superarm2005, Oktay, 4everRNA, pmitchell, vineetkosaraju, SteveWaters, Roysboy72, jennywren351, SonnyCoolMan, bradleesand, Jeaneaj, ErikViking, Shepard37, sesamestrong, Svenzie817, lazerepilasyon, peetereeter, danielMOFO, Filminfull, Daniela, DanaBeve, 77Tennifry, test720, bartzilla, babitan23, walkgraph, cocconic, kcoleman13, dinna0890, benlavi111, KZON, 5target, whereisbrian, quelia509, klctech, lighting2468, fixolasxd, penguinguy, sammex, addu02, indigopizza, samuel.russell31, mariiaamm, kurtnphoenix, AdditiveBook218, Colmeweb, MeganHansen, MochaLove, mave617, fherchov, karlbass36, cuukie_munster, ome, egale, david12345, dfvbrosh, docdan, Paaf, naseanh, crazyEyes, kilker101, Lidgered, blategameR, sohar, ugorur, PieThrower, tko, Hackathorn, Saiph, little light, Cleversy, ananas123456, mariella, 10102320, sahask, Dragonfire, Bonz, Nautilus567, mporter1, kingbenjamin, jenjen, riagad, cbond6590, MrnaRrnaTrna, Palms, SIVAGNANA, aliacoalwell, TrippleJ45, Majeed, jfoster, booby, dick2014, piotrek2023, seeen, spayne, Arena, xue, Jessi Jeffries, christo1, Skp43tc, rebeccaj, cyrilled, jocynicole, raginbrit, higiwili, KORZINGA, johnnie2038, ewilliampeterson, ac0852, Connor123, gatesfoundation-test, Alif_A, Domestic Chaos, Dash62g, r6preston, _SMG54_, thatginopeche, admicile32, DeviousCrow, rfc0005, machinelves, test7129, tekno4, Zhen_T, jwiswell, mikemass19, ZeroTheUltimate, moenco, PullJosh, itsallaboutmike, sostahl, hung10032002, Zimtelfe, Backpacker, PJG1996, lanzhu, mscowen, andrewdouch, andrevivi, test360, ohanwei, ashleyhamrick, achupacabra, test222, Grayseeroly, Noks19, susageP, ChatBot, dawidsimpsond, misbolsos, gjk27, mrf, Chath, alephn, TGerard, rudolphjosh, tlooms, framegrace, MarioLeao, test220, Rebeccareason1, xshagg, wuyehwen, Draksis45, darrin@yahoo.com, sanelrocio, mhd.eesa@gmail.com, banan120, Aiko_Eynos, bagnoll, alyssaking, gatesfoundation, bartozaur, Batou, ultimate4800, Akehebo, louban06, gottliebpet, rune3132, jazming11, John3-16, brofessorLyons, TheLovewaffe, HiddenSquid, SolarLiner, rchakraborty, Zesty Kumquat, Tristacular, kelseyfennewald, JosephSchwejk, soumi12345, adilrillon, Raszobs, pervyshev, heyshahboz, drwho42, Moonhaven, lira, Kevinpmac, diabeo, bnhump, Appollo, bob1234, rubescmar, ElonMusk, 16SpringCLauraVlacovsky, matou008, coljenkins, MASH, PearThreGreat, seanm, cvs.manas, alarosa, cadeboy28, kmcmenimen, Plasma2, borkpaladin, potato22, liliputien, Bwegs, TankyKiller, dhgghkjjhg, sauerl2793, Galaxspheria, Jeroen1602, laydylayne, Panda420, Dory fish123, chels1995, Brejo, LarryCuomo1126, Ageuke, JambonneauDorÈ, buttermilk23, Jarte, cssndrx, 10509i2103, DouglasBusa, Top10, amand13, krbillings, classdung, 8lie, Dwarow, Daddictif, YokiDiabeul, tomtom90, RVS doctor, miller1198, PReynor, book81able, BSimo, TWeston, Hawkey, Hoodielum, danielyim919, karentankist, bulha, poj4639, alysabuckler, derkarlotto, sara11111111111, iyess, klangen, selena.osman, Crookwood, cromaxis, bandlife, Davanint123, PhantomGryphon, Samuelada, AcruxYildun, Blacke3, pokilo, Rajan, live_and_laugh101, Eldar, mmhajla, DimitryM, Hettle, pandak, rflores23, nodnerb93, Osqi, maimas726, charlieg, Mike Reed, jcharles, applepie, Darkleader22, rarulu, StevenMelts, jero1987, tholcomb1, bullfrog, bdavis98, pinball wizard, adc04, Hoike808, loupi, Miketung, tastyteacup, henzo, aurumjd, NGzC, Matyas, kamdongibson, Seyedali, Kaz, ls3cam, mei ling, milky flynn, ashrestha4, tigerperr, Pixword, tdohert2, smitty3544, Park Soo-young, Eric Hamber, robi50, corrina1, ReapersEdge, stbo, Psycho, ribonucleic61, K9gaming, cconciatori, Brejen, SecretAgentNemo, bluebird1398, davidmcfreeman, Bubs, TrueSky, rykean33, braintwyster, zanzabar, Paivaan, Erikito, jschnabel735, goacego, Schamallo, coolkid12f, massimo, kylesun, Thursday, Suvidu, kp12345, Frucelle, sacausey, davidpat, chr2, testrookrd, kenzie_menzie, Strum, WesB, suzeterna, Quwertyn, tfinelli, ZAZOU207, ColMunchy, vluffy, dodo71, bubbamcbubs, procterronald, Amelie3, spikeydudey, mustardMan, trey8050, theangryunicorn, srdjan, joetalo, Abhilash, polark, rubarde, TheDawnDragon, noneedforaname, pandasecond, teejthenerd5, Maxis2000, tosina, 320123, sluke123, flixipix, Sango, mz7p40, cbao, edusoto, jorlando989, cheo, Ericlund19, dragonrenard, lydialydia, monkeygirl, stormAlchemist, dim90, Loogia, GabbyCeja, praveenjr, anbien, JamesKirk, walkedgraph122, NinjaNarwhal, eli.weaver04, automon, Meinew, solorofog, takpa, quasar28, RicardoR69odrigues, Cmcdo44, fcpway, ProgramALaAlex, Jannine, DankMcMeme, avzanzag, Koritsa, mbgilles, AlexanderdL, FrozenTux, gfb2003, Didier35, Johnson, than, reissfc, Leakybrains, TastyAngel, sophalex13, Donky, jelly123, Dimbas, mike33, luuuca, MeowsterHunter, dismalbogs, axedl, Ironeyeiris, andromeda14, spidermonkey, elicemck, UnsafeHurdle, ale, ???? ??????, little.dragon, bemljj, hanabanana, PColtman, mykaelapeterson17, gowachin, Amal A., Ms.Williams, bringing-midnight, tamas, UltimateNerd00, noooo, TraÔzer, Jorgelgq, olajka, froggy20, bekkel, Ginger1885, Stannum, SeaJayBravo982, BobÌ?ek, mandudeguy111, didi&aiai, dahfizz, DragonRanger, mikko114, eonur, wp19, bjohnson97211, walker01, adi2, arooj, Lupatran, williamp55, Fedian, grabas, lotus7productions, korshe, MasterPlasmide, renandf, wearefamily, Reyuen, barjos, ColeBianchi, clarkegar, cheercoder, magnuskri, L132465, richardrobinson, robymcbobster, intersysop, Butter3646, Chlorocidic, Jimroide, Shruti Dhara Sharma, alexis.lyles, guaninedestroyer, HopelessOptimist, cheepwine, jcallej0, Eva777, coolk, Loki-74, lucasmacielbiotech, francodanussi, davidprz, HAY SCIENCE, nachoninja, duskglow, xoxo_teddy, mebrou96, Shaheen, hiroman, LagnSteel, patrickmen11, theravin, RCHoss, Kenny01294, magyar, dell188, jonny4547, Exalaber, Sam_Ravenclaw, AhmedHexa, mistymountainminer, looool, Boss360, Lvenok, LittleFRU, king063, Trans-cendance, marygill, Milkshake, bryanlim, Raleigh.Billiot, AdrienneChan, harbir123, calculo, chuckdang, Nightingale_Royale, mayrablanco97, hjgf, SophiaTate, pentester101, Lord_Crakoz, holymedecine, Heidemac01, elementnumber46, humveev, OrigamiMarie, Schiffinor, arg1215, makashov_nurbol@mail.ru, fg, Camoes14, askro, xdperiod, Barik1931, TroyStopera, DOM31, Pinguim, tafos, Brent Schofield, alfmonc, Andrew4656, wipawadee, tweety0407, henryachan, carlosydos, IORNKING, HDBarbee, Justintoxicated, nickbooth, jp1224, HelloTheUniverse, glass130, joestudent, dbrown, Jdog, evilbiologist, Diogene, LucasAlmeida, Jai, mouseketia, jennifer24415, TruVenom, anurashrestha12, fapercin, JayFromNJ, acizkul58, couturin, ollieoe15, Jrbj2002, TitanPlayz, 12jp74, HScience, Fuzzybear, nfuente, qi4e2me1234q, blrosene, mtujamin, jbeletic, lraine, mrzwing, Byonzub, darchambault, gaminglazoorYT, Katiiie98, hickster, zundraim, EnigmaMXM, Infinite_Apollos, Didda, Folder1365, gabe.stokes, Stevan, Xavier11223, Firepuff0226, Gyrobotic, charizard325, woethe, maquique, coffevampire, Akrata111, Achraf112, stormclone80, JuliusHeinrich, cawolf, danielsundfeld, aaeeae, aaryanb, Gromtroll, jakovazor, thegamingfish, Aircondition, irimao, renocia, boi_social, silurio, DimaBiba, Lozy, Mr. Mollusk, Nofudecos, loumarylucy, ivg98, lupus_astartes, Louloudu44, Troll3, Kam, purzelbrot, Space Dolphin, paytn19, FriediFehlau, ZToRx, pokpok2710, the-pen, A churro8, Faisal, kobou, TheDoodleBanger, Dylanslegos, sivertz, katimars, orionmango, Tuberculosadeed, divisionbyzero, lmonti, SaraBot, PJ Kotze, Trystian, ViennaUCT, theeternabot, dtsaltas, Julianparengo
