# Supplementary figures and images for: EternaBrain: Automated RNA design through move sets and strategies from an Internet-scale RNA videogame
Source: PLoS Comput Biol. 2019 Jun 27;15(6):e1007059. doi: 10.1371/journal.pcbi.1007059 (PMC6597038; doi:10.1371/journal.pcbi.1007059)

6502989

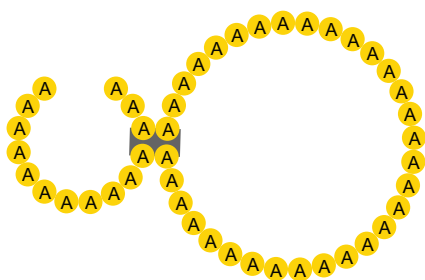

6502990

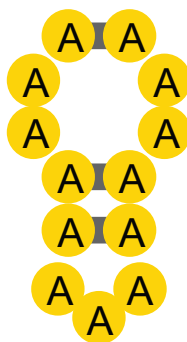

6502991

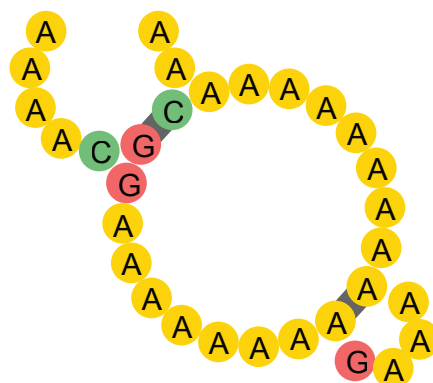

6502992

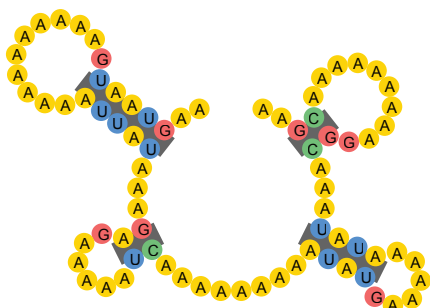

6502993

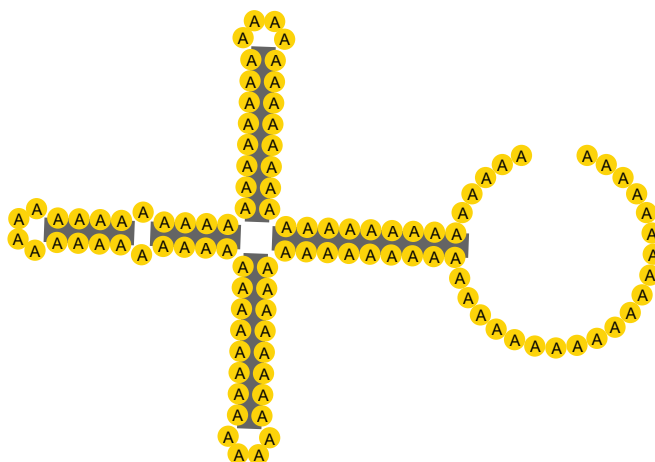

6502994

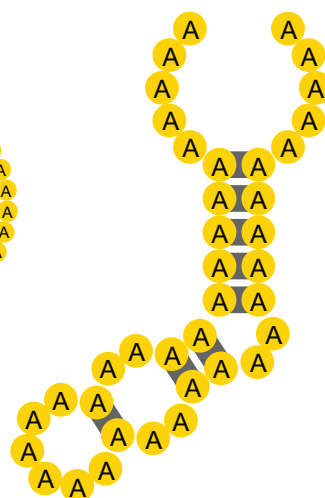

6502995

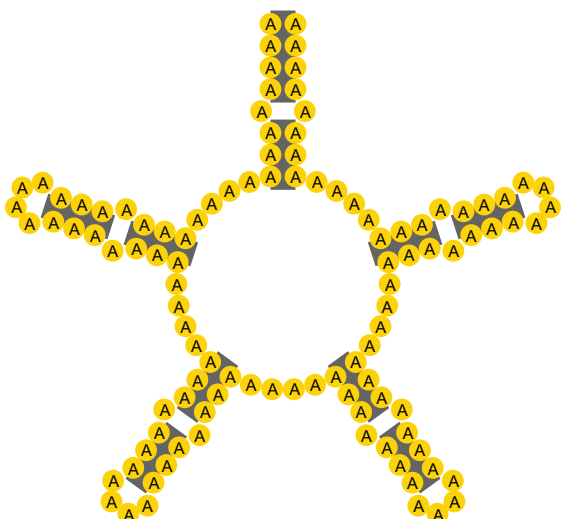

6502996

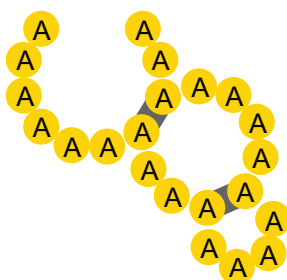

6502997

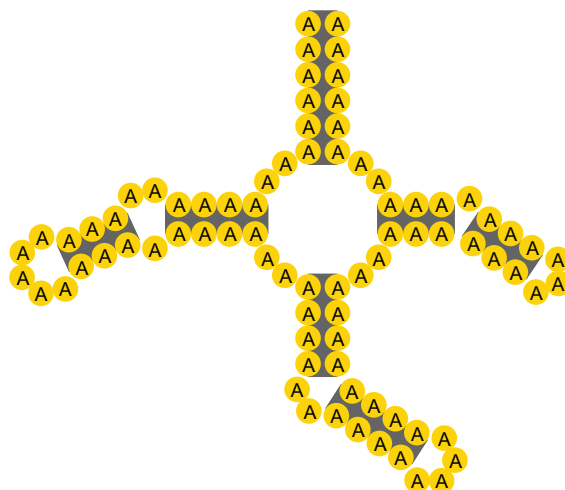

6502998

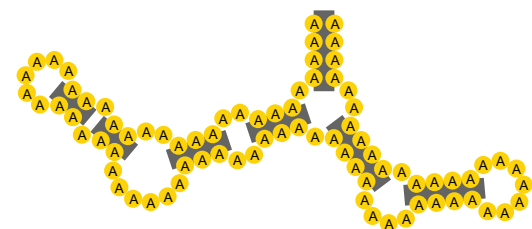

6502999

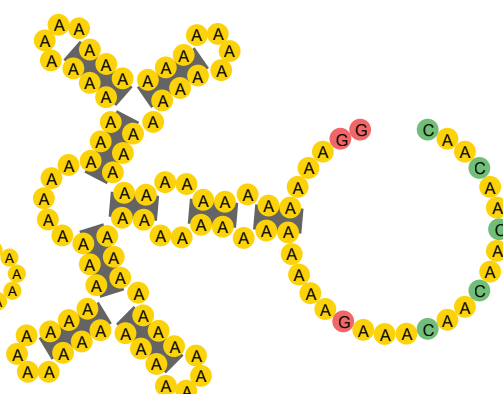

6503000

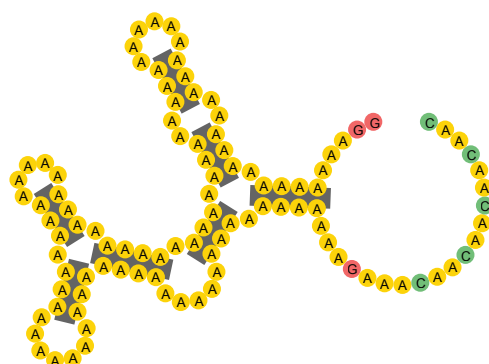

Supplement: S1 Fig — Most bases are adenine to represent the initial state of the puzzle before any mutations are made. Some bases that are not A represent “locked” bases which cannot be mutated. The 5’ end of each puzzle is at the top left, with the puzzle drawn counter-clockwise from that point. (PDF) [file pcbi.1007059.s002.pdf]

470862

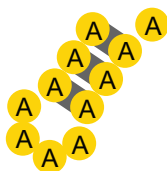

924481

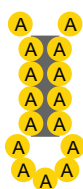

969610

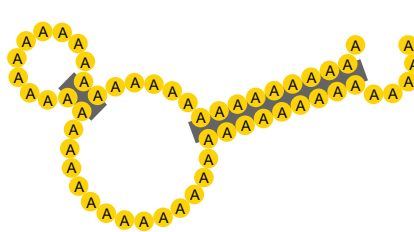

969616

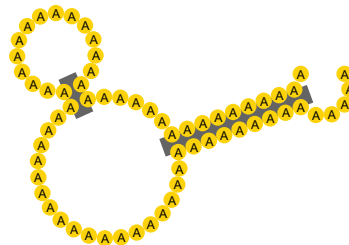

970889

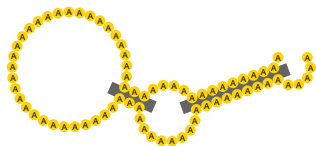

972995

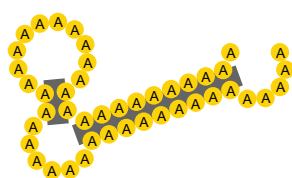

973010

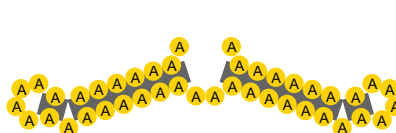

977775

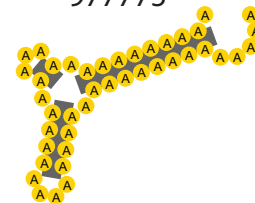

988108

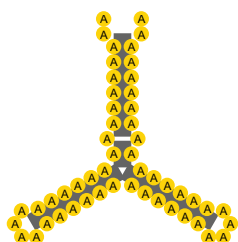

1005186

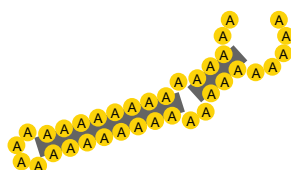

1021173

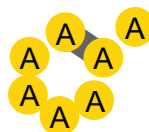

1074756

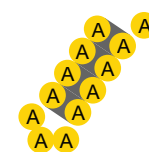

2173229

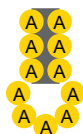

2361236

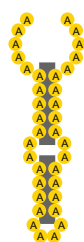

2361348

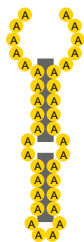

2438008

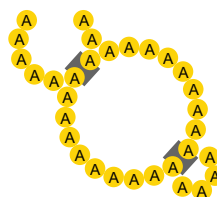

2440447

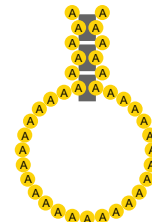

2442301

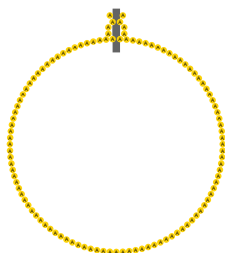

2586215

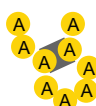

2817913

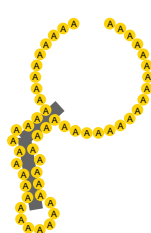

2826515

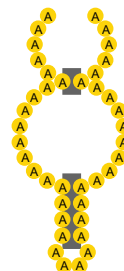

2836984

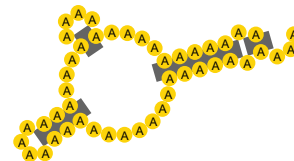

2859701

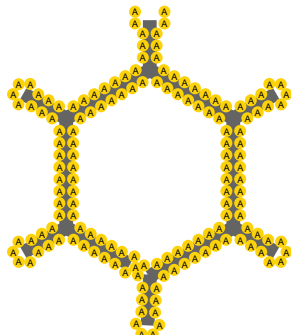

2863060

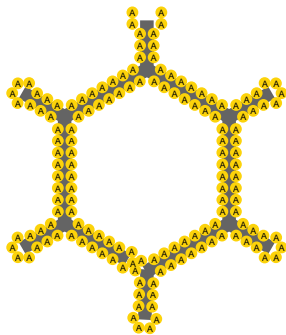

2904837

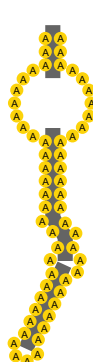

3124709

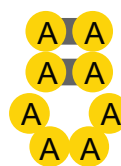

3149691

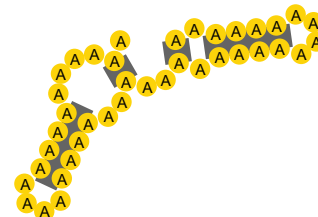

3174768

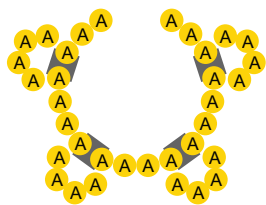

3177248

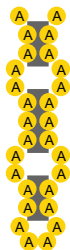

3245898

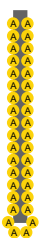

3251484

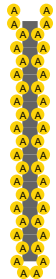

3294054

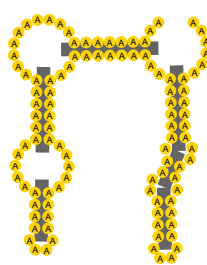

3450464

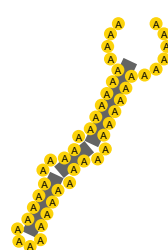

3450485

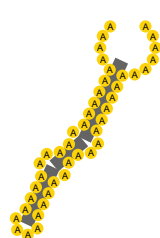

3468526

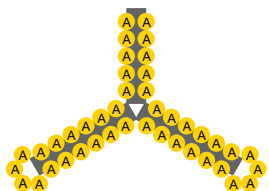

3468547

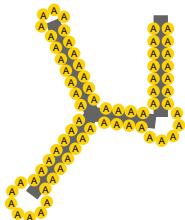

3475185

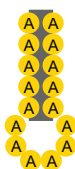

3484458

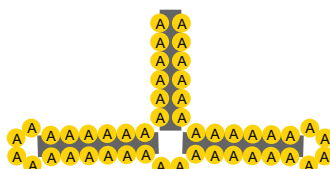

3500158

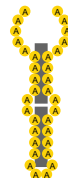

3522605

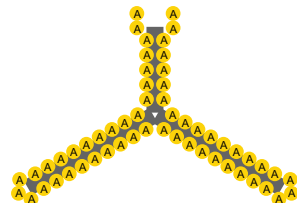

3522647

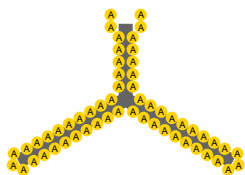

3536334

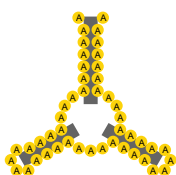

3536373

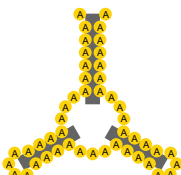

3536444

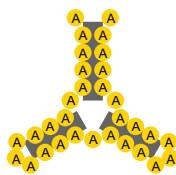

3542351

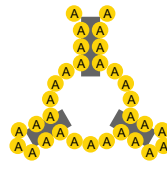

3633213

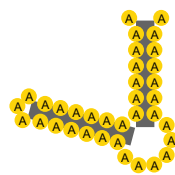

3634228

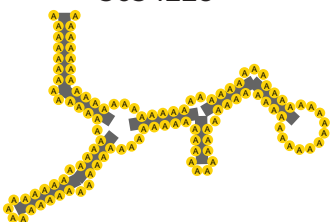

3634635

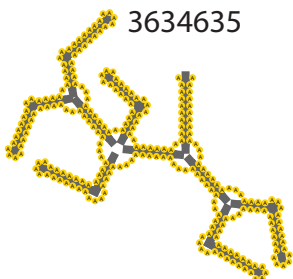

3682204

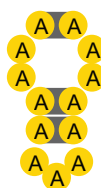

3682239

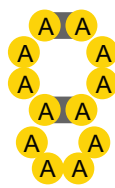

3704385

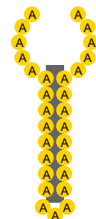

3704388

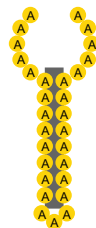

3704391

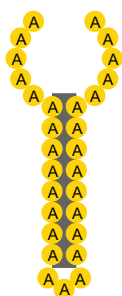

3857631

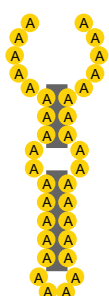

3912739

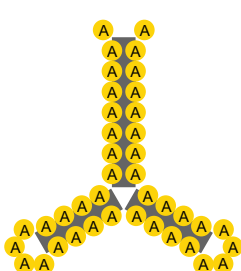

3951241

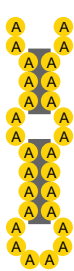

3951286

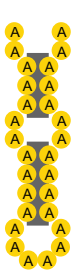

3951333

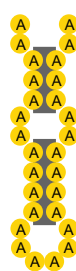

4024177

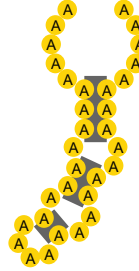

4062836

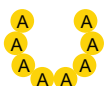

4255265

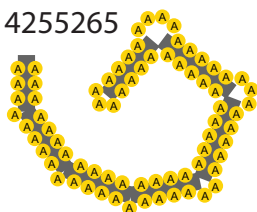

4258598

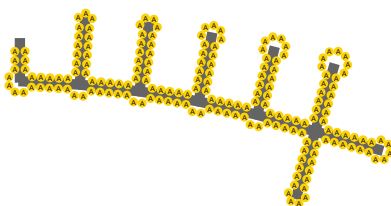

4263778

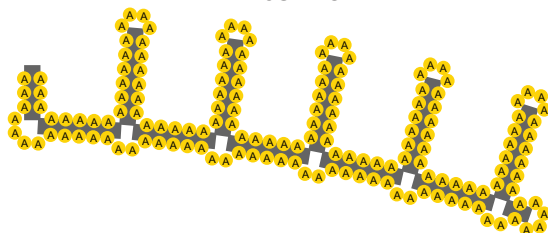

4265757

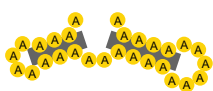

4292000

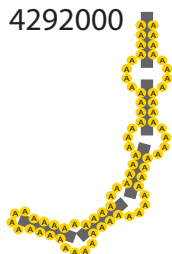

4312727

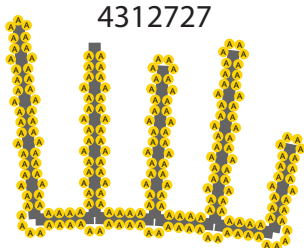

4561479

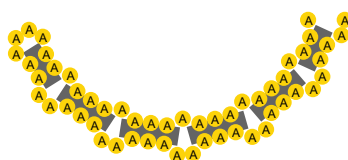

4960718

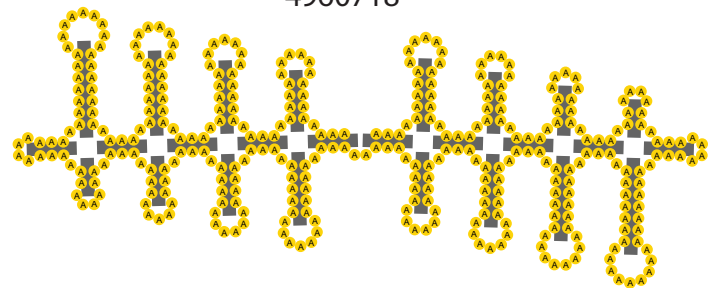

5353710

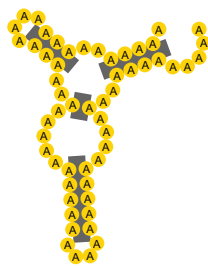

5359220

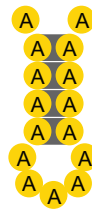

5721020

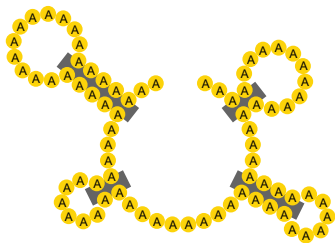

5723240

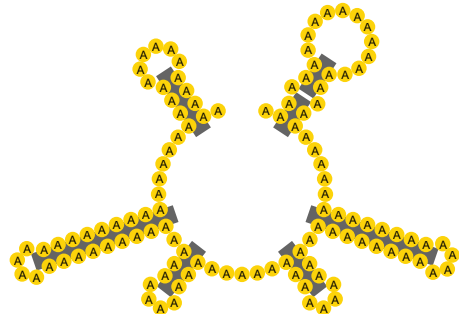

5797240

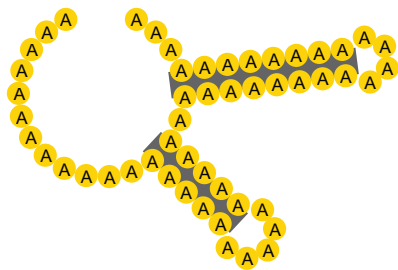

6396259

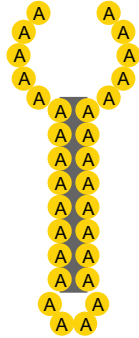

Supplement: S2 Fig — Some puzzle structures are repeated because the locked bases (bases which cannot be mutated) are different for the two puzzles. The 5’ end of each puzzle is at the top left, with the puzzle drawn counter-clockwise from that point. (PDF) [file pcbi.1007059.s003.pdf]

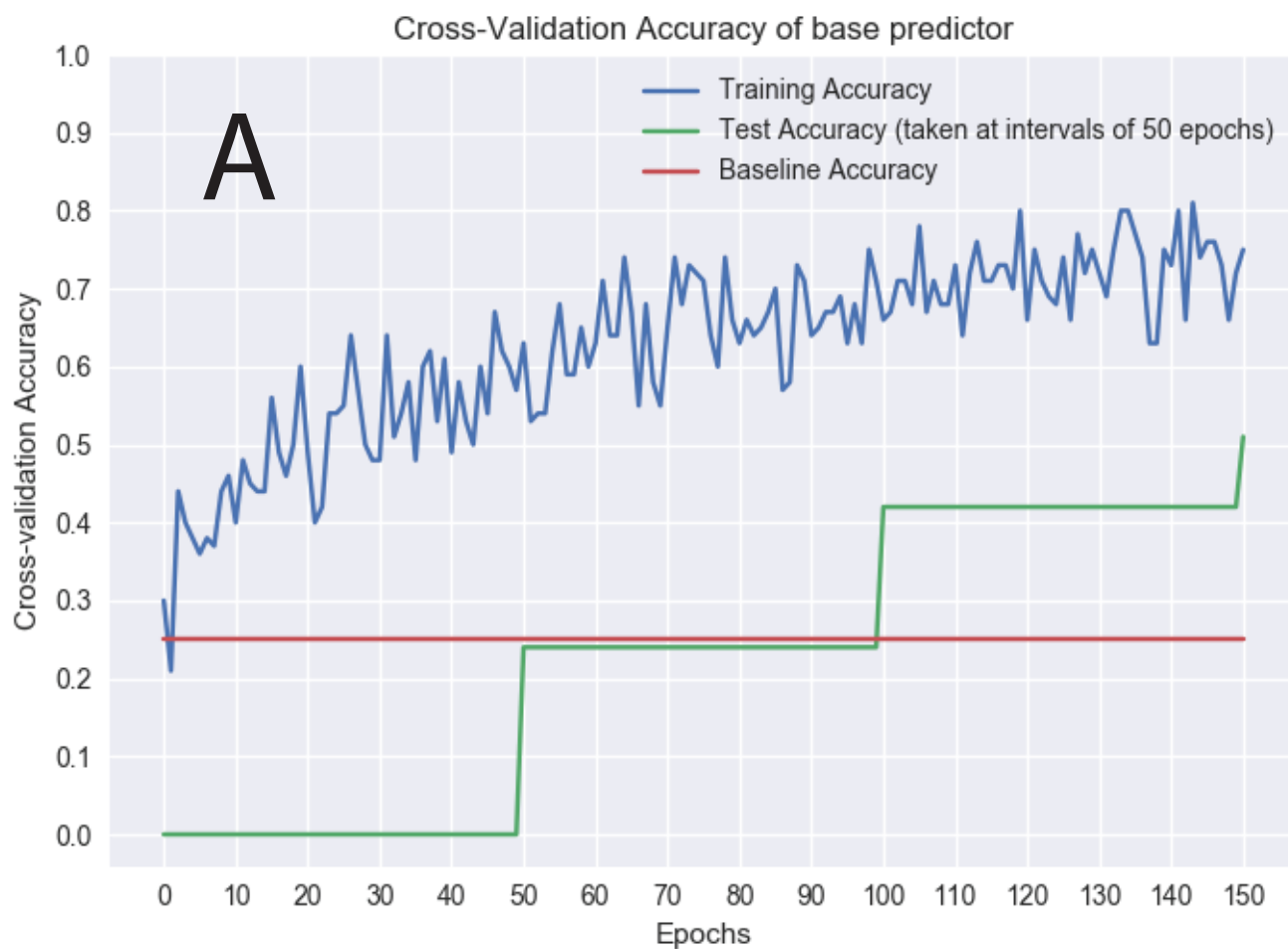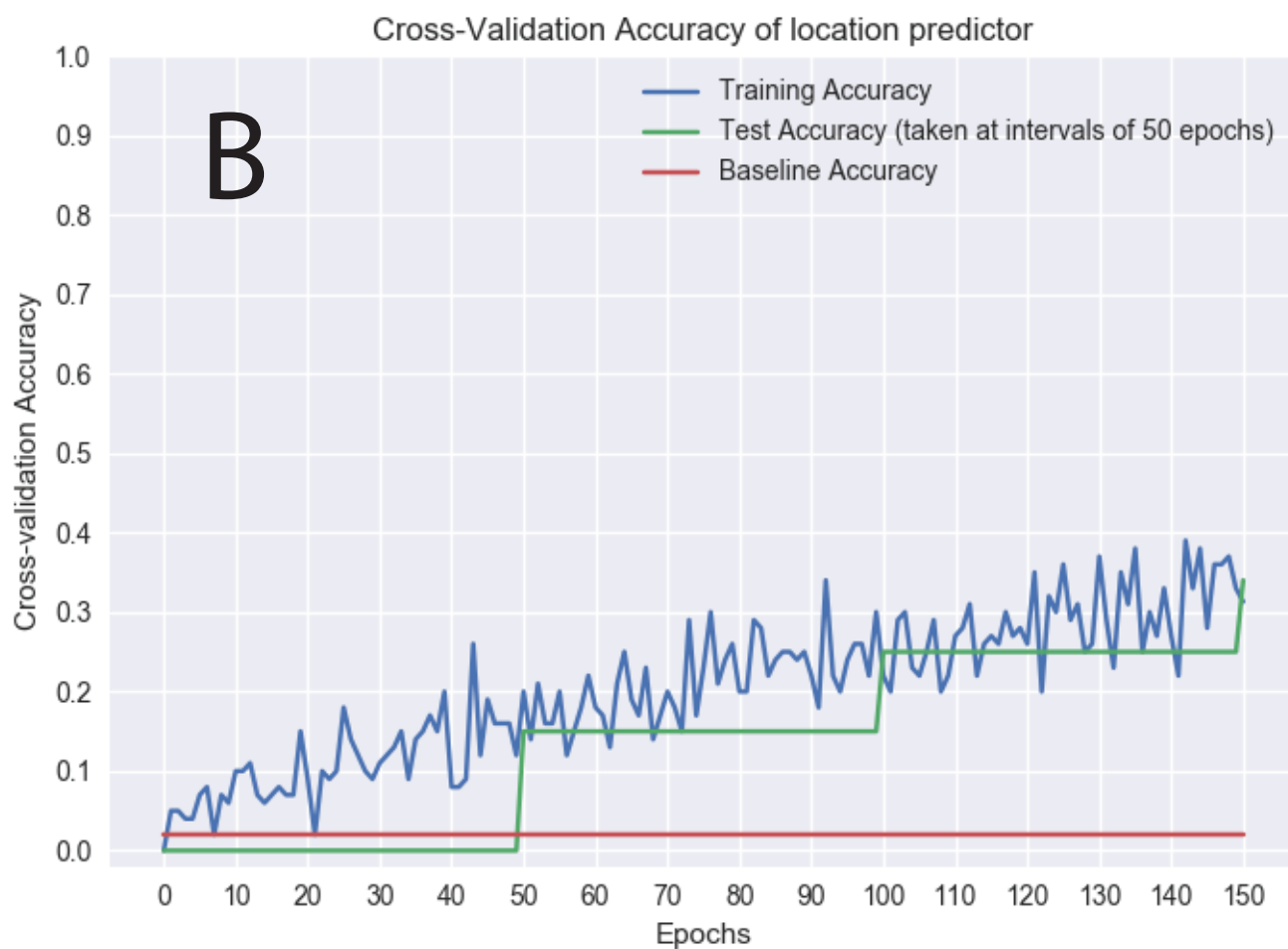

Supplement: S3 Fig — (A) Cross-validation accuracy of location predictor per epoch of training. (B) Cross-validation accuracy of base predictor per epoch of training. An epoch of training is one complete pass of backpropagation, retraining, and receiving feedback of performance over all 30,447 solutions (305 mini-batches of 100 solutions each). (PDF) [file pcbi.1007059.s004.pdf]

Time needed to solve a puzzle of a given length

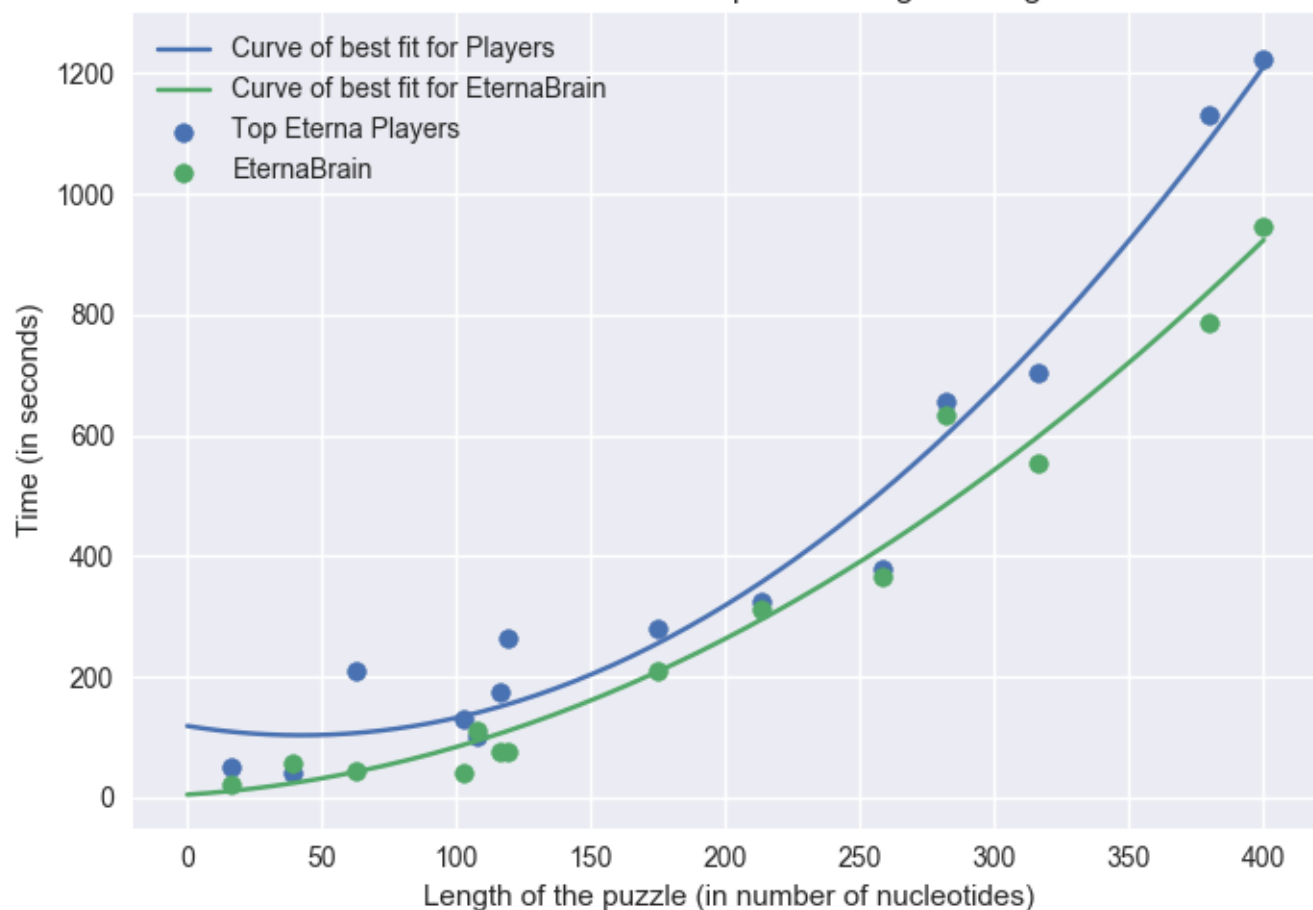

Moves needed to solve a puzzle of a given length

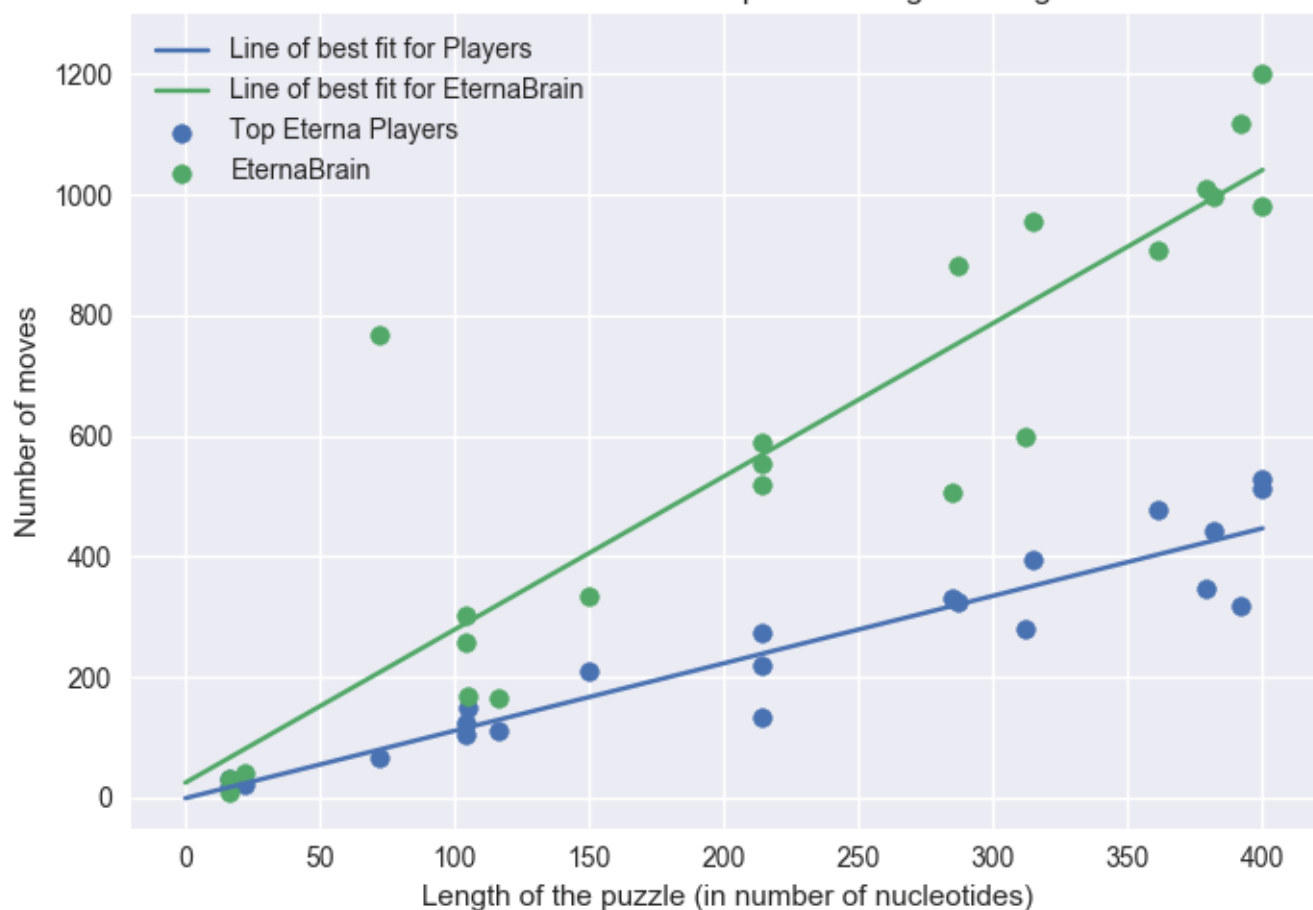

Supplement: S4 Fig — (A) Median time and (B) number of moves needed to solve Eterna100 puzzles. Puzzles that were only solvable by Eterna human players are not shown. (PDF) [file pcbi.1007059.s005.pdf]
