# Supplementary material for: EternaBrain: Automated RNA design through move sets and strategies from an Internet-scale RNA videogame
Source: PLoS Comput Biol. 2019 Jun 27;15(6):e1007059. doi: 10.1371/journal.pcbi.1007059 (PMC6597038; doi:10.1371/journal.pcbi.1007059)
Supplement: S1 Table — (DOCX) [file pcbi.1007059.s006.docx]

**Supporting Table S1.** **Hyperparameter search for neural net architecture.**

| Model Hyperparameters^a^ | Base Train Accuracy^b^ | Base Test Accuracy^b^ | Location Train Accuracy^b^ | Location Test Accuracy^b^ |
| --- | --- | --- | --- | --- |
| 0.1 dropout, Adam, sigmoid, 10 conv layers, 4 fc layers, CNN | 0.50 | 0.34 | 0.10 | 0.021 |
| 0.0 dropout, Adam, sigmoid, 10 conv layers, 4 fc layers, CNN | 0.61 | 0.26 | 0.15 | 0.018 |
| 0.0 dropout, Adam, relu, 10 conv layers, 4 fc layers, CNN | 0.33 | 0.30 | 0.05 | 0.03 |
| 0.1 dropout, Adam, Sigmoid, 10 fc layers, DNN | 0.30 | 0.24 | 0.03 | 0.01 |

^a^ We trained 4 neural networks on the *eternamoves-large* dataset. We trained two different types of neural networks: a standard feed-forward deep neural network and a convolutional neural network. We varied a number of different hyperparameters, including dropout, number of neurons per layer, number of layers, optimizer function (Adam, SGD, etc.), activation function (relu, hyperbolic tangent, sigmoid).

^b^Accuracy for best model trained on *eternamoves-large*.
