# Supplementary material for: EternaBrain: Automated RNA design through move sets and strategies from an Internet-scale RNA videogame
Source: PLoS Comput Biol. 2019 Jun 27;15(6):e1007059. doi: 10.1371/journal.pcbi.1007059 (PMC6597038; doi:10.1371/journal.pcbi.1007059)
Supplement: S2 Table — (DOCX) [file pcbi.1007059.s007.docx]

**Supporting Table S2.** **Number of solutions and moves for top players used in *eternamoves-select*.**

| **Rank**^a^ | **Usernames** | **IDs** | **Num_solutions** | **Num_moves** |
| --- | --- | --- | --- | --- |
| **0** | mat747 | 267 | 6666 | 926857 |
| **1** | akhyatt | 1623 | 183 | 11989 |
| **2** | machinelves | 2577 | 2 | 21 |
| **3** | lroppy | 2804 | 1949 | 161644 |
| **5** | tommyd | 4375 | 2723 | 204442 |
| **6** | Eli Fisker | 8627 | 8032 | 2141064 |
| **7** | starryjess | 11775 | 97 | 1707 |
| **8** | armin | 19442 | 855 | 33768 |
| **9** | Brourd | 24263 | 1018 | 244522 |
| **10** | Meechl | 26574 | 313 | 9732 |
| **13** | c-quence | 29762 | 589 | 25905 |
| **14** | stevetclark | 32487 | 241 | 7494 |
| **15** | jandersonlee | 32627 | 2277 | 106044 |
| **16** | choo | 34334 | 232 | 6255 |
| **17** | drake178 | 34596 | 158 | 4194 |
| **19** | hoglahoo | 36921 | 1632 | 107248 |
| **20** | fluffy3 | 39309 | 56 | 1717 |
| **23** | 77Tennifry | 42101 | 2 | 26 |
| **25** | JR | 42833 | 4706 | 618972 |
| **27** | Jsci | 43776 | 2144 | 284749 |
| **29** | wawan151 | 44191 | 255 | 39711 |
| **30** | macclark52 | 44631 | 22 | 1164 |
| **31** | hotcreek | 46281 | 6 | 118 |
| **32** | jnicol | 48166 | 241 | 11290 |
| **33** | Jieux | 48170 | 4216 | 698491 |
| **34** | Tesla'sDisciple | 50065 | 1588 | 86498 |
| **35** | Malcolm | 52361 | 5478 | 740470 |
| **37** | RedSpah | 55082 | 3 | 83 |
| **39** | janetmason | 56579 | 435 | 14565 |
| **40** | garydfisher | 57654 | 797 | 41876 |
| **41** | Omei | 57675 | 6136 | 426299 |
| **42** | wateronthemoon | 57743 | 882 | 38259 |
| **43** | cataway | 57874 | 63 | 1943 |
| **44** | Hyphema | 58224 | 57 | 9943 |
| **45** | rnjensen45 | 60391 | 10 | 260 |
| **47** | theravin | 61244 | 2 | 7 |
| **49** | AndrewKae | 64544 | 1135 | 161290 |
| **52** | Gres | 77611 | 498 | 52423 |
| **53** | ulfang | 78801 | 866 | 51782 |
| **55** | Zanna | 87216 | 1309 | 49302 |
| **57** | salish99 | 132701 | 67 | 657 |
| **58** | biggestlegoheroicafanever | 133043 | 415 | 31166 |
| **59** | wookietank | 136909 | 576 | 17795 |
| **60** | VirgieP3 | 143547 | 106 | 2889 |
| **62** | Mayanne | 152013 | 379 | 9998 |
| **63** | benrh | 179978 | 6510 | 565822 |
| **65** | skyblue | 207838 | 2061 | 188398 |
| **66** | Marzena11 | 209752 | 240 | 9534 |
| **67** | Eized | 216078 | 321 | 19185 |
| **68** | dl2007 | 223393 | 1800 | 1773005 |
| **69** | joy45 | 225023 | 1062 | 49752 |
| **70** | DeNa | 231977 | 6991 | 949354 |
| **71** | cynwulf28 | 233206 | 5798 | 545017 |

^a^ Participant rank is based on total number of puzzles solved on Eterna at the time of the study, and not just puzzles in *eternamoves-select*; not all participants solved puzzles chosen for *eternamoves-select*.
